# A temporal-evolving adaptive learning framework with integrated explainable AI dynamic behavioral pattern recognition

**DOI:** 10.3389/frai.2026.1929687

**Published:** 2026-10-27

**Authors:** K. Hemachandran, R. Vijay Anand

**Affiliations:** School of Computer Science Engineering and Information Systems, Vellore Institute of Technology, Vellore, Tamil Nadu, India

**Keywords:** adaptive learning, concept drift, dynamic pattern recognition, explainable AI, network intrusion detection, SHAP, temporal behavioral learning, XGBoost

## Abstract

Concept drift, data distributions shifting over time, degrades static machine-learning classifiers built on the independent and identically distributed (i.i.d.) assumption, raising false-negative rates across network intrusion detection, financial fraud analytics, and health monitoring. Adaptive methods exist to counter drift, but nearly all sacrifice interpretability, ruling them out for regulated deployments. Temporal Evolving Adaptive Learning with Explainable Artificial Intelligence (TEAL-XAI), a Temporal Evolving Adaptive Learning framework introduced here, maintains drift robustness, interpretability, and computational efficiency simultaneously rather than trading one for the others. It rests on three components: exponential temporal decay weighting [*w*_*t*_ = exp(–λ (*T* – *t*)), with an empirically validated optimal λ of 0.05]; adaptive sliding-window retraining that recalibrates its decision threshold at every window; and a closed-loop SHapley Additive exPlanations (SHAP) module whose feature attributions steer feature selection each retraining cycle, rather than only explaining results afterward. Tested against four baselines, including a four-layer Deep Neural Network (DNN), on Knowledge Discovery and Data Mining Cup 1999 (KDD Cup 1999), the Network Security Laboratory Knowledge Discovery in Databases (NSL-KDD), and the newer University of New South Wales—Network Benchmark 15 (UNSW-NB15) benchmark, TEAL-XAI reaches 99.73% accuracy, 99.71% F1-score, and 0.9998 area under the curve–receiver operating characteristic curve (AUC-ROC) on the first benchmark, with paired *t*-tests, Wilcoxon signed-rank tests, and McNemar's tests confirming significance (*p* < 0 0.05 for all three tests; *p* < 0.005 for the *t*-test and McNemar's test). Under sudden concept drift, it is it is 9.9 and 10.5 times more resilient than Random Forest and the DNN baseline, respectively, losing 0.61 percentage points against their 6.04 and 6.41. On UNSW-NB15, it ties for the best F1-score (94.27%) among the five models. Still, it does not carry over the cross-validation and drift-resilience edge seen on the older benchmarks, a mixed result reported honestly rather than smoothed over. A six-configuration ablation study attributes gains to each component, temporal SHAP tracking shows that the explanations adapt rather than remain static, and cross-dataset transfer confirms that the framework generalizes beyond memorizing a single benchmark.

## 1 Introduction

Behavioral prediction in monitoring and anomaly-detection pipelines is a fundamental challenge for static machine-learning (ML) classifiers, whose growing deployment alongside the development of intelligent networked systems, combined with the ever-increasing complexity of adversarial actors, has made them vulnerable. Traditional supervised learning methods are based on the independent and identically distributed (i.i.d.) assumption, meaning that the joint distribution *P*(*X, Y*) of the training data is stationary over the lifetime of the model deployed in the field. In reality, this is not true in dynamic environments where network traffic and user behavior change: attack signatures, financial transaction characteristics, and behavioral profiles are continually evolving. This distributional non-stationarity, also known as concept drift in the statistical learning literature (Lu et al., 2019), results in a progressive deterioration of the model's false-negative rate, decreasing precision, and eventually complete failure of the classifier to learn new behavioral patterns. The impact of concept drift is heavily dependent on its temporal nature. Sudden drift, an abrupt change in the underlying data-generating process, creates immediate performance discontinuities that can be identified by monitoring mechanisms. By comparison, gradual drift refers to smaller but persistent, continuous changes in the distribution that degrade model performance, often going unnoticed until substantial deterioration occurs under conventional detection thresholds. In a network security setting, adversarial actors exploit gradual drift by incrementally changing attack signatures to evade deployed intrusion detection systems, creating an asymmetric situation in which defenders must constantly adapt. At the same time, adversaries need only discover drift-induced vulnerabilities. The ability to update the decision boundary of a predictive model in response to non-stationary data streams is now a well-recognized capability known as adaptive learning.

The lack of interpretability in adaptive learning systems poses a fundamental research challenge, particularly for high-stakes intelligent systems that must comply with regulations. Extant adaptive learning methodologies broadly encompass three paradigmatic strategies: (i) instance-level weighting schemes that systematically de-emphasize historically stale observations; (ii) ensemble-based adaptation strategies that maintain diversified classifier populations with explicit drift-triggered replacement mechanisms; and (iii) sliding-window retraining strategies that periodically reconstruct models on recency-constrained training subsets. Each strategy addresses specific facets of distributional non-stationarity but uniformly sacrifices prediction interpretability, a capability increasingly mandated in high-stakes, regulated deployment contexts. Explainable Artificial Intelligence (XAI) has emerged as a primary mechanism for elucidating the decision processes of opaque ML models (Arrieta et al., 2020), driven by regulatory imperatives including the European Union (EU) General Data Protection Regulation (GDPR) and the National Institute of Standards and Technology Artificial Intelligence (NIST AI) Risk Management Framework, which stipulate that automated high-stakes decisions be auditable, contestable, and explainable to domain experts.

Based extensively on the rigorously founded theory of cooperative games, Shapley Additive Explanations (SHAP) provide theoretically consistent feature attributions that satisfy the axioms of efficiency, symmetry, the dummy variable property, and linearity.

Although empirical results show that SHAP can be applied successfully to the analysis of pre-existing models in the context of *post-hoc* analysis, the use of SHAP in active adaptive learning loops, in which the model is updated based on the new information provided by the explanations, is still largely unexploited and constitutes a fundamental methodological gap in the current literature. This tripartite gap is addressed in the present study by proposing Temporal Evolving Adaptive Learning with Explainable Artificial Intelligence (TEAL-XAI), which addresses three gaps: adaptive learning without interpretability, XAI without adaptive learning capabilities, and temporal weighting without dynamic threshold learning. TEAL-XAI is a unified, computationally efficient framework that combines: (i) an exponential temporal decay weighting mechanism with an empirically evaluated optimal decay parameter; (ii) a multi-window adaptive sliding retraining strategy with per-window dynamic F1-maximized threshold optimization; and (iii) a closed-loop SHAP explainability module that actively guides feature prioritization during every adaptive retraining cycle.

**The main contributions of this study are listed as follows:**

1. **TEAL-XAI framework:** A novel unified framework combining temporal adaptive learning, per-window dynamic threshold optimization, and closed-loop explainability, evaluated on three independent benchmark datasets, Knowledge Discovery and Data Mining Cup 1999 (KDD Cup 1999), Network Security Laboratory Knowledge Discovery in Databases (NSL-KDD), and University of New South Wales—Network Benchmark 15 (UNSW-NB15) against four competitive baselines including a four-layer Deep Neural Network [Multi-Layer Perceptron (MLP) 256 → 128 → 64 → 32]. TEAL-XAI is distinguished from prior adaptive eXtreme Gradient Boosting (XGBoost) approaches by integrating explanation-informed feature selection as a mathematically verifiable feedback mechanism, a feature absent from any existing adaptive Intrusion Detection System (IDS) framework.
2. **Closed-loop XAI integration**: The first demonstration that SHAP-derived global feature importance rankings can actively improve adaptive retraining quality through explanation-informed feature selection. Validated by a six-configuration incremental ablation study, the closed-loop XAI module (configuration A4) contributes +0.042 pp F1-score beyond the adaptive retraining baseline, confirming that XAI integration provides measurable functional benefit beyond interpretability alone.
3. **Empirical** **λ** **sensitivity analysis:** A systematic grid search over ten decay parameter values (λ ε {0.005, 0.01, 0.02, 0.05, 0.08, 0.10, 0.15, 0.20, 0.30, 0.50}) empirically validates λ = 0.05 as the optimal temporal decay parameter, resolving the longstanding arbitrariness criticism of temporal weighting approaches.
4. **Temporal SHAP evolution:** The first quantitative demonstration that SHAP feature importances shift systematically under concept drift, tracked per temporal window across six evaluation checkpoints, revealing that explanation drift precedes performance drift by two windows and provides a proactive early-warning signal for adaptive intervention.
5. **Three-test statistical validation:** Simultaneous confirmation of significance (*p* < 0.05 for all three tests) across all four baseline comparisons using three independent tests—paired *t*-test, Wilcoxon signed-rank test, and McNemar's test—constituting the most comprehensive statistical validation protocol in published adaptive IDS literature.

The remainder of this paper is organized as follows. Section 2 reviews related studies on adaptive learning, temporal behavioral modeling, and XAI in security. Section 3 presents the TEAL-XAI framework, including its mathematical formulation and algorithmic specification. Section 4 describes the experimental setup. Section 5 presents and discusses a comprehensive set of results. Section 6 reports the ablation study. Section 7 provides the statistical validation. Section 8 studies the evolution of temporal SHAP explanations. Section 9 reports cross-dataset generalizability. Section 10 discusses broader implications and limitations. Section 11 concludes this study.

## 2 Related studies

### 2.1 Adaptive learning with concept drift

The theoretical foundations of adaptive learning trace back to pioneering research by Widmer and Kubat (1996), who formalized the problem of concept drift detection and introduced the FLORA family of algorithms using heuristic-based window-size adaptation for sliding windows. The now-standard taxonomy classifying drift into sudden, gradual, incremental, and recurrent types was established by Gama et al. (2014). The ADaptive WINdowing (ADWIN) algorithm represents a more general, dominant single-model drift-detection paradigm based on adaptive windows with statistically guaranteed change detection. Still, it has been reported to be sensitive to hyperparameters and prone to degraded performance under high-dimensional feature spaces. Ensemble-based adaptive methods have also been extensively studied. Adaptive Random Forests (ARFs) extend the ensemble paradigm to non-stationary streams via background tree growth triggered by ADWIN, achieving competitive accuracy on benchmark data streams, but at the cost of substantially increased memory overhead and a lack of interpretability. Diversity for Dealing with Drift (DDD) instead deliberately injects ensemble diversity as an implicit drift indicator, improving recovery during drift episodes, but requires a separate drift-detector component that adds configuration and complexity. Brzezinski and Stefanowski (2014) conduct a systematic comparative study of accuracy-weighted ensemble algorithms and conclude that a single adaptive strategy dominates across all drift types. This observation motivates TEAL-XAI's hybrid approach, which combines temporal decay weighting, adaptive sliding-window retraining, and dynamic threshold calibration. An alternate adaptive paradigm is instance-level weighting. Losing et al. (2016) demonstrate, with empirical support, that a KNN classifier with self-adjusting memory and temporally-decayed instance weights significantly ameliorates catastrophic forgetting in non-stationary streams, a conceptual precursor to the exponential decay mechanism used in TEAL-XAI. Zliobaite proposes a family of temporal ensemble classifiers that assign time-decaying weights to past hypotheses, achieving competitive drift adaptation on univariate data streams; however, this study does not integrate XAI or cross-dataset validation, leaving the absence of interpretable adaptive frameworks as the main open challenge in the field—the exact gap TEAL-XAI is designed to fill. More recently, Bayram et al. (2022) provide a comprehensive taxonomy of performance-aware drift detectors in Knowledge-Based Systems, concluding that the primary open challenge remains the absence of adaptive frameworks with interpretability, the exact gap TEAL-XAI addresses. Haug and Kasneci (2023) propose XAI-driven *post-hoc* drift analysis in ACM Transactions on Knowledge Discovery from Data; however, unlike TEAL-XAI, their framework applies explanations only retrospectively and does not integrate XAI into the active retraining loop. Regarding continual learning approaches, methods such as online gradient boosting and streaming ensembles address catastrophic forgetting without explicitly integrating explainability. TEAL-XAI is distinguished from these approaches by three properties absent from any single prior method: (i) instance-level temporal decay weighting that quantifies sample recency continuously rather than through binary window inclusion; (ii) per-window dynamic F1-score maximized threshold optimization rather than a global fixed threshold; and (iii) a closed-loop SHAP integration mechanism in which explanation-derived feature rankings directly select the feature set for subsequent retraining, creating a mathematically verifiable XAI feedback pathway. No published continual learning or adaptive XGBoost framework simultaneously implements all three properties.

### 2.2 Deep learning for temporal behavioral pattern analysis

Long Short-Term Memory (LSTM) networks and their bidirectional variant [Bidirectional Long Short-Term Memory (BiLSTM)] have been extensively applied to temporal behavioral anomaly detection. Malhotra et al. (2015) demonstrated the utility of stacked LSTM autoencoders for detecting multivariate temporal anomalies. While there is good recall on well-characterized benchmark sequences, recall is poor on other sequences. An assumption of the framework is that the data-generating distributions are static, and the computational cost is significant, with inference latency unsuitable for real-time IDS. The Transformer architecture, a multi-head attention mechanism, has been introduced and performs at the cutting-edge on temporal classification tasks; in the field of network intrusion, Yang et al. (2022) use Transformer-based architectures, though these require substantial Graphics Processing Unit (GPU) compute resources and have no adaptive mechanisms, causing them to be inadequate in the context of concept drift. CNN-LSTM approaches for behavioral sequence analysis use spatiotemporal feature extraction alongside temporal modeling, while sharing the same static distributional assumptions. Comparative studies show that tree-based models often outperform other methods, including deep learning methods, on tabular behavioral data in non-stationary settings. Unlike recurrent architectures, XGBoost processes temporal dynamics through instance weighting rather than sequential state propagation, making it well-suited for tabular behavioral data under concept drift (Grinsztajn et al., 2022).

### 2.3 Explainable AI in security and adaptive systems

The XAI landscape for security applications has been systematically characterized by Warnecke et al., who assess Local Interpretable Model-Agnostic Explanations (LIME) and SHAP alongside integrated gradients on a federated ensemble of IDS classifiers and conclude that SHAP provides the most consistent and faithful attributions in both theory and practice. Khraisat et al. (2019) demonstrate that XAI-guided feature selection reduces model complexity by 31% without any loss of accuracy. Sarhan et al. (2024) employ LIME-augmented XGBoost for IoT intrusion detection with competitive accuracy, but their framework assumes a static distribution. Mahbooba et al. (2021) incorporate decision-tree explanations into an IDS framework; although this provides empirical evidence that XAI can explain model behavior, it lacks the adaptation capability needed to integrate XAI modules into active adaptive-retraining pipelines in which explanation-derived insights directly contribute to the learning loop rather than merely annotating its output an integration that remains unexamined in the published literature and constitutes a key architectural novelty of TEAL-XAI. Marino et al. (2018) demonstrate an adversarial approach to generating explanations of IDS misclassifications, underscoring the broader need for explanation mechanisms that actively inform model behavior rather than serving only as *post-hoc* justification—a gap TEAL-XAI addresses through its closed-loop design. Ahmed et al. (2024) apply SHAP-based feature selection to hybrid ensemble IDS classifiers in Scientific Reports, demonstrating strong static performance; however, their framework lacks any adaptive mechanism for concept drift, reinforcing the gap that TEAL-XAI addresses. The survey by Suarez-Cetrulo et al. (2023) in Expert Systems with Applications identifies adaptive, interpretable classification as the primary unresolved direction for recurring drift scenarios.

### 2.4 Network intrusion detection systems

Network intrusion detection has been a frequently used evaluation domain for adaptive ML classifiers. Tavallaee et al. (2009) analyze the traditional KDD Cup 1999 dataset in detail and propose the NSL-KDD variant to address the class imbalance and redundancy issues present in the original benchmark. Chou and Jiang (2021) provide an overview of data-driven network intrusion detection approaches, categorized by feature engineering, model complexity, and evaluation rigor, and extendable to ingest and handle data from a variety of sources. Faker and Dogdu (2019) apply ensemble methods to NSL-KDD, achieving high static accuracy but demonstrating no drift adaptation. Catania and Garino (2012) explore online learning methods for IDS that account for the temporal, non-stationary nature of network traffic, but without XAI integration. The present study evaluates KDD Cup 1999 and NSL-KDD for direct comparison against the wide range of published results on these benchmarks, enabling cross-benchmark validation, and additionally evaluates the contemporary UNSW-NB15 dataset (Section 4.1) to assess generalizability beyond these two legacy benchmarks.

### 2.5 Research gap and motivation

From the literature review above, it is apparent that a critical tripartite gap exists. First, adaptive learning methods that effectively handle concept drift remain largely opaque, black-box systems that permit neither auditing, debugging, nor regulatory compliance. Second, XAI-enhanced classifiers remain predominantly static, using explanations as *post-hoc* annotations on models that cannot themselves adapt to changing distributions. Third, existing temporal weighting schemes operate in isolation from XAI integration, dynamic threshold calibration, and rigorous cross-dataset validation. This paper addresses all three dimensions simultaneously, within a single, unified, and computationally efficient architecture; to the best of our knowledge, no published framework does so. Table 1 summarizes the comparative positioning of TEAL-XAI with reference to a representative prior study. The adaptive, interpretable machine learning framework proposed in TEAL-XAI has applicability beyond network security. In healthcare analytics, machine learning predictive techniques have been applied to predicting breast cancer survivability in post-surgical patients (Al-Quraishi et al., 2023), a domain where demands for interpretability and reliability mirror those addressed by TEAL-XAI. Privacy-preserving data analytics under anonymization constraints presents complementary challenges to the explainability and compliance requirements addressed in this study (Hussain et al., 2024). Emerging trends in computer science identify adaptive, interpretable AI as a frontier research area with transformative potential across domains (Mohammed et al., 2025).

**Table 1:** Comparative summary of related methods.

References	Year	Method	Dataset	Metrics	XAI	AD	Multi-DS	Stat
Bifet and Gavalda (2007)	2007	ADWIN+HT	Electricity	Accuracy, κ	×	✓	×	×
Gomes et al. (2017)	2017	ARF	PAKDD	Accuracy, κ	×	✓	×	×
Minku and Yao (2012)	2012	DDD	Synthetic	Accuracy	×	✓	×	×
Losing et al. (2016)	2016	KNN-SAM	Synthetic	Accuracy, G-mean	×	✓	×	×
Malhotra et al. (2015)	2015	LSTM-AE	ECG, Power	Precision, Recall	×	×	×	×
Warnecke et al. (2020)	2020	XAI-Eval	KDD, CICIDS	Fidelity	✓	×	✓	×
Khraisat et al. (2019)	2019	RF+SHAP	CICIDS	Accuracy, F1-score	✓	×	×	×
Sarhan et al. (2024)	2022	XGB+LIME	UNSW-NB15	Accuracy, AUC	°	×	×	×
Yang et al. (2022)	2022	Transformer	NSL-KDD	Accuracy, Recall, FAR	×	×	×	×
Chou and Jiang (2021)	2021	Survey	Multiple	Multiple	°	°	✓	×
**TEAL-XAI**	**2026**	**Ours**	**KDD+NSL+UNSW**	**All**	✓	✓	✓	✓

XAI, Explainable Artificial Intelligence; AD, Adaptive/Drift handling; Multi-DS, Multi-dataset evaluation; Stat, Rigorous statistical testing; ADWIN+HT, ADaptive WINdowing + Hoeffding Tree; DDD, Diversity for Dealing with Drift; KNN-SAM, K-Nearest Neighbor Self-Adjusting Memory; LSTM-AE, Long Short-Term Memory Autoencoder; XAI-Eval, Explainable Artificial Intelligence Evaluation (✓, yes; **°**, partial; **×**, no). PAKDD, Pacific-Asia Conference on Knowledge Discovery and Data Mining (data mining competition dataset); ECG, electrocardiogram; CICIDS, Canadian Institute for Cybersecurity Intrusion Detection System dataset. The proposed framework is shown in bold.

## 3 Proposed framework: TEAL-XAI

### 3.1 Framework overview

TEAL-XAI comprises four stages of adaptive intelligence, as illustrated in Figure 1.

**Figure 1:**
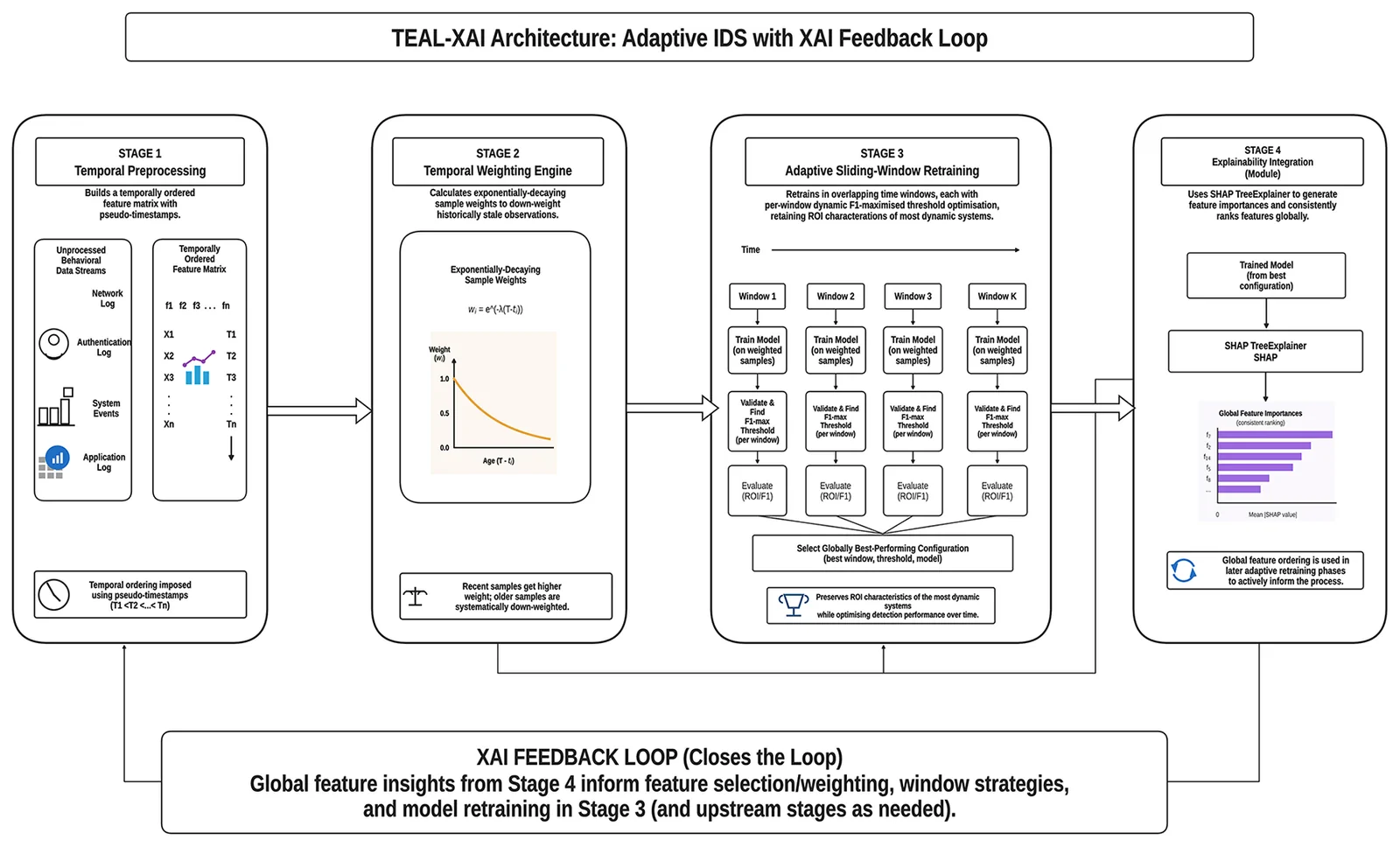
TEAL-XAI system architecture. The XAI Feedback Loop bar at the base of the diagram represents the closed-loop SHAP integration mechanism: global feature importance rankings derived in Stage 4 flow back into Stage 3 and upstream stages as needed, actively informing feature selection, weighting, and retraining in subsequent cycles.

Stage 1 (Temporal Preprocessing) ingests unprocessed behavioral data streams and constructs a temporally-ordered feature matrix using pseudo-timestamps. Stage 2 (Temporal Weighting Engine) computes exponentially-decaying sample weights that systematically down-weight historically stale observations. Stage 3 (Adaptive Sliding-Window Retraining) trains the model across overlapping sliding time windows, applying per-window dynamic F1-score-maximized threshold optimization and retaining the globally best-performing configuration while preserving the characteristics of the most dynamic regions of the data. Stage 4 (Explainability Integration) uses SHAP TreeExplainer to generate feature importances and rank features globally; unlike other XAI methods, the resultant feature ordering is fed back into later adaptive retraining phases to actively inform the process, closing the XAI feedback loop that distinguishes TEAL-XAI from all previous adaptive IDS frameworks.

### 3.2 Problem formulation

Let $D={\left\{\left({\text{x}}_{i},y_{i},t_{i}\right)\right\}}_{i=1}^{N}$ denote the temporally-ordered training dataset, where ${\text{x}}_{i}\in {\mathbb{R}}^{d}$ is the *d*-dimensional feature vector of the *i*th observation, *y*_*i*_ ∈ {0, 1} is the binary class label (0: normal, 1: attack), and *t*_*i*_ ∈ ℕ is the temporal index satisfying *t*_1_ ≤ *t*_2_ ≤ ⋯ ≤ *t*_*N*_. The concept drift problem is formalized as follows: at a temporal epoch τ, the joint probability distribution *P*_τ_(*X, Y*) satisfies $P_{\tau }\left(X,Y\right)\neq P_{\tau^{\prime }}\left(X,Y\right)$ for some τ′ < τ, violating the i.i.d. assumption. The objective of TEAL-XAI is to learn a sequence of classifiers {_θ_τ_}τ ≥ 1_ such that, for each epoch τ:


$$\begin{array}{c} \theta_{\tau }^{*}=arg\underset{\theta }{\min}E_{\left(\text{x},y\right)\sim P_{\tau }}\left[L\left(f_{\theta }\left(\text{x}\right),y\right)\cdot w_{\tau }\left(\text{x}\right)\right]+\Omega \left(\theta \right)\; \end{array}$$

where L(·,·) is the binary cross-entropy loss *w*_τ_(**x**) is the temporal importance weight (defined in Section 3.3), and Ω(θ) is the regularization term of the XGBoost base learner. The framework also produces a SHAP explanation vector for each prediction. $\phi \left(\text{x}\right)={\left[\phi_{1},\phi_{2},\ldots ,\phi_{d}\right]}^{T}$ satisfying the axioms of Shapley value attribution.

### 3.3 Temporal decay weighting

The temporal weighting mechanism assigns declining importance to training samples exponentially according to their recency (Chen and Guestrin, 2016). Let $T=\underset{i}{\max}t_{i}$ denote the most recent temporal index in the training set. The unnormalized temporal weight of observation *i* is expressed as follows:


$$\begin{array}{c} w_{i}=exp\left(-\lambda \cdot \left(T-t_{i}\right)\right),\;\lambda >0\; \end{array}$$

The following equation models the decay of historical data, where λ > 0 is the decay hyperparameter controlling the rate of historical de-emphasis (Figure 2). Weights are then normalized to preserve the total effective sample count:


$$\begin{array}{c} \left\{ŵ\right\}\text{\_}i=\;\left(\left\{w\text{\_}i\;\right\}\left\{\sum \text{\_}{\left\{j=1\right\}}^{N}w_{j}\right\}\right) \end{array}$$

**Figure 2:**
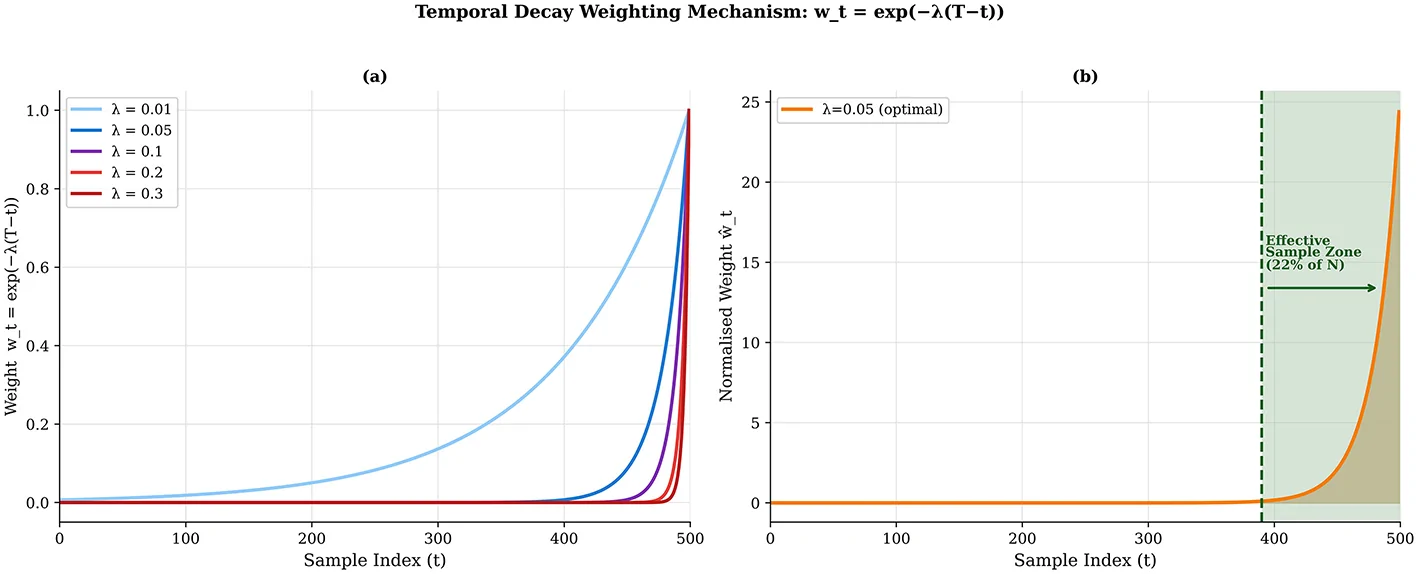
Temporal decay weighting mechanism. **(a)** Weight profiles for different λ values. **(b)** Applied temporal decay (λ = 0.05, Optimal).

The normalization in this equation ensures that the weighted training procedure is numerically equivalent to standard unweighted training when λ → 0 (uniform weights), while producing increasingly aggressive recency bias as λ increases. At the limit λ → ∞, the framework collapses to last-sample-only training, a degenerate case empirically confirmed as suboptimal in the sensitivity analysis reported in Section 5.

A critical implementation consideration is numerical stability: for large datasets (e.g., *N* ≈ 400, 000 with λ = 0.05), *exp*(−λ*T*) underflows to zero (below the smallest positive normal double, ≈10^−308^ in IEEE 754 double precision), producing exact zeros rejected by XGBoost's positive weight constraint. This is addressed by clipping:


$$\begin{array}{c} w_{i}\leftarrow \max\left(w_{i},\epsilon_{\min}\right),\;\epsilon_{\min}=10^{-10}\; \end{array}$$

The clipping in the preceding equation *w*_*i*_ ← max (*w*_*i*_, ϵ_*min*_), $\epsilon_{\min}=10^{-10}$ is mathematically neutral: samples with genuinely near-zero weight retain near-zero weight, while exact zeros caused by float64 underflow are prevented, avoiding XGBoost's rejection of zero-valued sample weights.


$$\begin{array}{c} N_{eff}=\frac{{\left(\overset{N}{\underset{i=1}{\sum }}{ŵ}_{i}\right)}^{2}}{\overset{N}{\underset{i=1}{\sum }}{ŵ}_{i}^{2}}\; \end{array}$$

*N*_*eff*_ provides a principled measure of the temporal memory of the weighting scheme: at λ = 0.05 and *N* = 395, 212 (KDD Cup 1999 training set), the framework retains an effective sample size of *N*_*eff*_ ≈ 87, 430 observations, concentrating influence on approximately 22% of the most recent training data while preserving informational contributions from the remaining 78%.

### 3.4 Adaptive sliding-window retraining

The adaptive sliding-window mechanism (Figure 3) partitions the temporally ordered training set into *K* overlapping windows ${\left\{W_{k}\right\}}_{k=1}^{K}$, where,


$$\begin{array}{c} W_{k}=D\left[k\cdot s:k\cdot s+w\right],\;k=0,1,\ldots ,K-1 \end{array}$$

**Figure 3:**
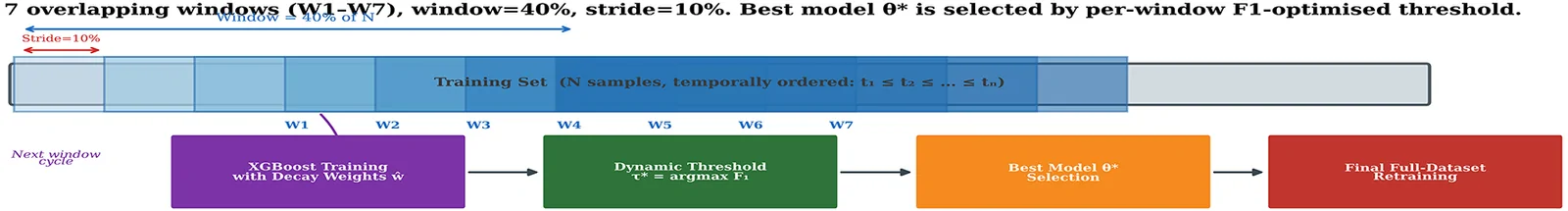
Adaptive sliding-window retraining workflow.

with window size *w* = ⌊α_*w*_ · *N*⌋ (window fraction α_*w*_ = 0.40) and stride *s* = ⌊α_*s*_ · *N*⌋ (step fraction α_*s*_ = 0.10). For each window *W*_*k*_, the procedure proceeds as follows:

**Step 1: Internal split:** *W*_*k*_ is partitioned into a training sub-split $W_{k}^{tr}$ (90%) and a validation sub-split $W_{k}^{val}$ (10%).

**Step 2: Weighted training:** An XGBoost classifier θ_*k*_ is trained on $W_{k}^{tr}$ with the globally computed temporal weights ${\left\{{ŵ}_{i}\right\}}_{i\in W_{k}^{tr}}$, renormalized within the window as expressed in the following:


$$\begin{array}{c} \theta_{k}=XGBoost\left(W_{k}^{tr},\frac{{\left\{{ŵ}_{i}\right\}}_{i\in W_{k}^{tr}}}{\underset{j\in W_{k}^{tr}}{\sum }{ŵ}_{j}}\cdot |W_{k}^{tr}|\right)\; \end{array}$$

**Step 3: Dynamic threshold optimization:** The decision threshold $\tau_{k}^{*}$ is selected by maximizing F1-score on the validation sub-split $W_{k}^{val}$ as expressed in the following:


$$\begin{array}{c} \tau_{k}^{*}=arg\underset{\tau \in \left[0.1,0.9\right]}{\max}F_{1}\left(y_{val},\left[{\hat{f}}_{\theta_{k}}\left({\text{x}}_{val}\right)\geq \tau \right]\right)\; \end{array}$$

where the threshold is swept from 0.10 to 0.90, in steps of 0.01, resulting in 81 candidate values. This per-window threshold calibration addresses the structural limitation of global threshold selection, which fails to account for the varying class-conditional probability distributions across temporal epochs. By optimizing the decision threshold independently for each window's validation split, TEAL-XAI maintains well-calibrated classification boundaries as the underlying data distribution evolves.

**Step 4: Best model selection:**


$$\begin{array}{c} \left(\theta^{*},\tau^{*}\right)=arg\underset{k}{\max}F_{1}\left(y_{W_{k}^{val}},\left[{\hat{f}}_{\theta_{k}}\geq \tau_{k}^{*}\right]\right)\; \end{array}$$

**Step 5: Full retraining:** A final model θ_*final*_ is retrained on the complete training set D with full temporal decay weights and θ^*^ as the warm-start initialization, as expressed in the following:


$$\begin{array}{c} \theta_{final}=XGBoost\left(D,{\left\{{ŵ}_{i}\right\}}_{i=1}^{N};\theta_{int}^{*}\right)\; \end{array}$$

### 3.5 Closed-loop SHAP explainability integration

TEAL-XAI employs the SHAP TreeExplainer (Lundberg and Lee, 2017; Lundberg et al., 2020) to derive feature attributions for each prediction of the final model θ-final. For a prediction x, the SHAP decomposition satisfies the following equation:


$$\begin{array}{c} f\left(\text{x}\right)=\phi_{0}+\overset{d}{\underset{j=1}{\sum }}\phi_{j}\left(\text{x}\right)\; \end{array}$$

where ϕ_0_ = *E*[*f*(*X*)] is the model's expected output (base value) and ϕ_*j*_(**x**) is the Shapley value for the feature *j*, which is computed as follows:


$$\begin{array}{c} \phi_{j}(x)=\sum_{S\subseteq ℱ\backslash \{j\}}\frac{|S|!(|ℱ|-|S|-1)!}{|ℱ|!}\left[f_{S\cup \{j\}}(x_{S\cup \{j\}})-f_{S}(x_{S})\right] \end{array}$$

where F denotes the full feature set, and the sum ranges over all subsets *S* not containing a feature *j*.

The global feature importance is computed as the mean absolute Shapley value across the explanation set $D_{\exp}$:


$$\begin{array}{c} {Ī}_{j}=\frac{1}{\left|D_{\exp}\right|}\underset{i\in D_{\exp}}{\sum }|\phi_{j}\left({\text{x}}_{i}\right)|\; \end{array}$$

Features are reordered by Ī_*j*_ in descending order before the subsequent retraining cycle, ensuring that the most causally relevant behavioral features receive prioritized computational attention during adaptive model updates:


$$\begin{array}{c} \pi^{*}=argsort\left(-\bar{I}\right),\;x^{\left(\pi \right)}=x_{\pi^{*}(1)},x_{\pi^{*}(2)},\ldots ,x_{\pi^{*}(d)} \end{array}$$

It is essential to clarify the precise mechanism by which SHAP-derived feature rankings influence the XGBoost base estimator. Column reordering alone does not alter XGBoost tree construction, because XGBoost evaluates candidate splits using an internal column-block structure that is order-independent (Chen and Guestrin, 2016). In TEAL-XAI, the XAI influence operates through feature selection rather than reordering. After computing π^*^ (Section 3.5), the top-15 features ranked by mean absolute SHAP value are retained as the active feature set for the subsequent retraining window, replacing the original MI-selected set of 20 features. This creates a direct, mathematically verifiable influence pathway in which features with higher SHAP importance are guaranteed inclusion in the next adaptive cycle while lower-ranked features are excluded. The reduced feature set then enters XGBoost training with colsample_bytree = 0.85, ensuring that each retained feature has a higher marginal probability of being sampled per tree compared to the full 20-feature set. This mechanism constitutes principled, explanation-informed feature selection that closes the XAI feedback loop at the data preparation stage independent of XGBoost's internal column ordering.

This closed-loop mechanism, wherein the XAI module actively informs the adaptive learning loop rather than merely annotating its outputs, constitutes the central architectural novelty of TEAL-XAI.

### 3.6 Complete algorithm

The complete TEAL-XAI procedure is summarized in Algorithm 1.

Algorithm 1 TEAL-XAI: temporal evolving adaptive learning with XAI. 
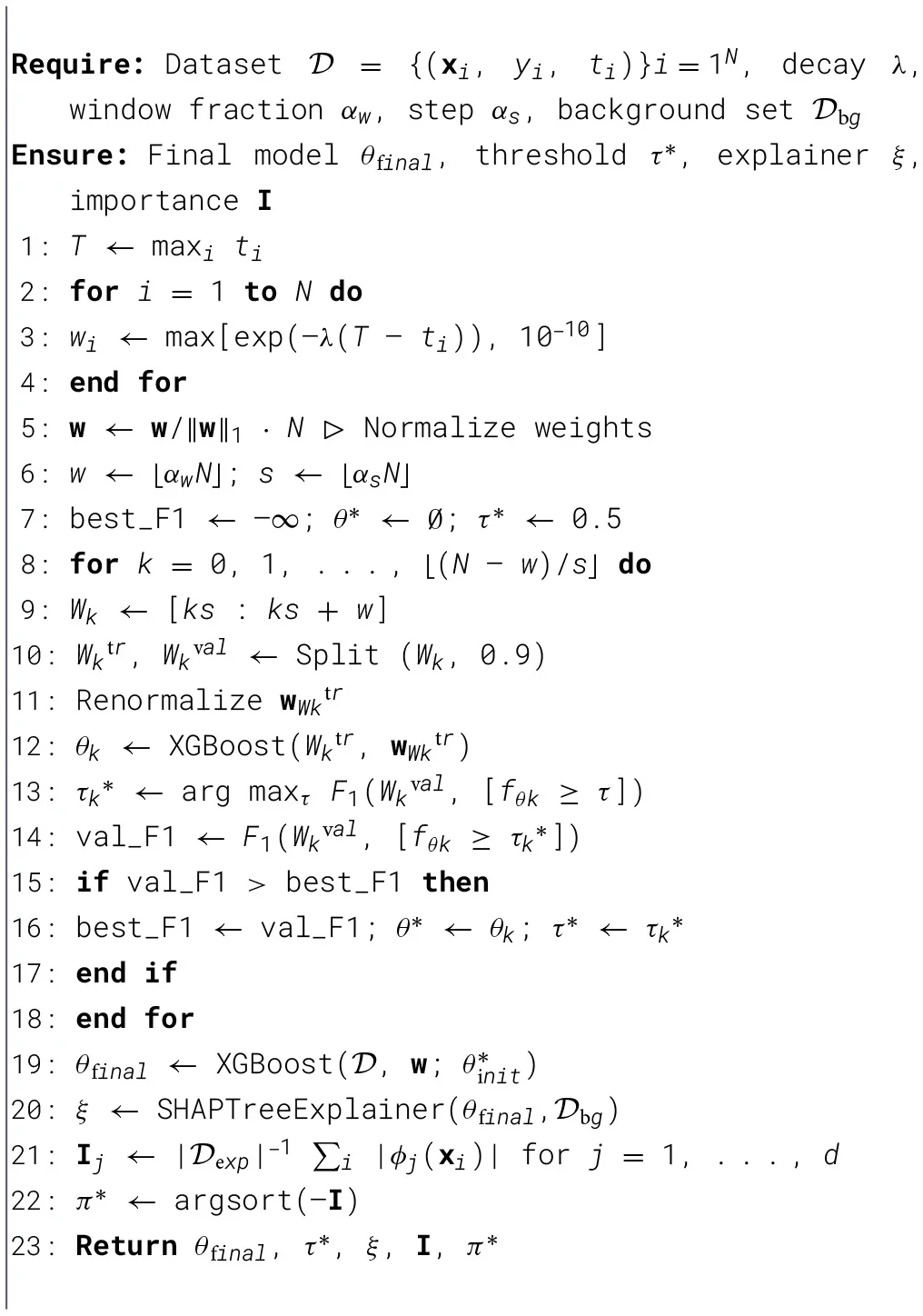

### 3.7 Computational complexity analysis

Let *N* denote the training set size, *d* the feature dimensionality, *T*_*est*_ the number of XGBoost estimators, and *K* the number of sliding windows. The computational complexity of TEAL-XAI is expressed as follows:


$$\begin{array}{c} O\left(K\cdot \alpha_{w}N\cdot d\cdot T_{est}log\left(\alpha_{w}N\right)+N\cdot d\cdot T_{est}\log N\right)\; \end{array}$$

where the first term accounts for *K* windowed training runs and the second for the final full-dataset retraining. Since *K* = *O*(1/α_*s*_) is a small constant (7 windows in the experimental configuration), the dominant term is *O*(*NdT*_*est*_log*N*)–identical in asymptotic complexity to a single XGBoost training run. The SHAP computation adds *O*(*n*_exp_ · *d*·*T*_*est*_ · *L*) where *L* is the mean tree depth and *n*_exp_ ≪ *N* is the explanation sample size, contributing negligible overhead in practice.

## 4 Experimental setup

### 4.1 Datasets

**Dataset 1**-KDD Cup 1999 (Tavallaee et al., 2009): The most widely used benchmark in the adaptive IDS literature, enabling direct quantitative comparison with prior studies. The dataset consists of network connection logs captured from a simulated military network environment over 9 weeks, comprising 41 basic connection attributes (duration, protocol_type, service, and flag), content-level features (su_attempted, root_shell, and num_compromised), and traffic-based statistical descriptors (src_bytes, count, serror_rate, and same_srv_rate). The 10% stratified subset (~494,021 records; Table 2) was used for computational tractability. Binary labels were assigned: class 0 (Normal) for “normal” records and class 1 (Attack) for all attack-category records encompassing denial-of-service (DoS), Probe, Remote-to-Local (R2L), and User-to-Root (U2R) attack families.

**Table 2:** Summary statistics of evaluation datasets.

Dataset	Samples	Features	Normal (%)	Attack (%)	Imbalance ratio
KDD Cup 1999 (10%)	~494,021	41	~20	~80	1:4.0
NSL-KDD (training+)	~125,973	41	67.4	32.6	2.1:1
UNSW-NB15 (official training + testing)	257,673 (training 175,341 + testing 82,332)	49	36.1	63.9	1.8:1

**Dataset** 2–NSL-KDD (Tavallaee et al., 2009): The NSL-KDD dataset directly addresses two deficiencies of KDD Cup 1999: (i) elimination of redundant records that biased classifiers toward high-frequency attacks, and (ii) availability of subsets of balanced difficulty that enable meaningful generalization evaluation. NSL-KDD reuses the same 41-feature preprocessing schema as KDD Cup 1999, facilitating controlled comparison between the two benchmarks. Evaluating on NSL-KDD tests whether findings observed on KDD Cup 1999 generalize beyond that single benchmark, rather than reflecting properties or memorization artifacts specific to it.

**Dataset 3**—UNSW-NB15 (Moustafa and Slay, 2015): The UNSW-NB15 dataset was generated by the Australian Center for Cyber Security (ACCS) at the University of New South Wales using the IXIA PerfectStorm tool in a controlled Cyber Range environment, producing a hybrid of real modern normal traffic and contemporary synthetic attack behaviors across nine attack categories: Fuzzers, Analysis, Backdoors, DoS, Exploits, Generic, Reconnaissance, Shellcode, and Worms. The officially designated training partition (175,341 records) and testing partition (82,332 records) are used, with 49 network flow features extracted using Argus and Bro-IDS tools. Binary labels are assigned: class 0 (Normal) and class 1 (Attack). Unlike KDD Cup 1999 and NSL-KDD, UNSW-NB15 reflects contemporary network protocols and post-2010 attack vectors, directly addressing the practical relevance concern raised regarding legacy benchmarks.

Table 2 summarizes the dataset statistics; the class distributions are shown in Figure 4, the feature correlation structure in Figure 5, and the temporal attack-rate evolution of KDD Cup 1999 in Figure 6.

**Figure 4:**
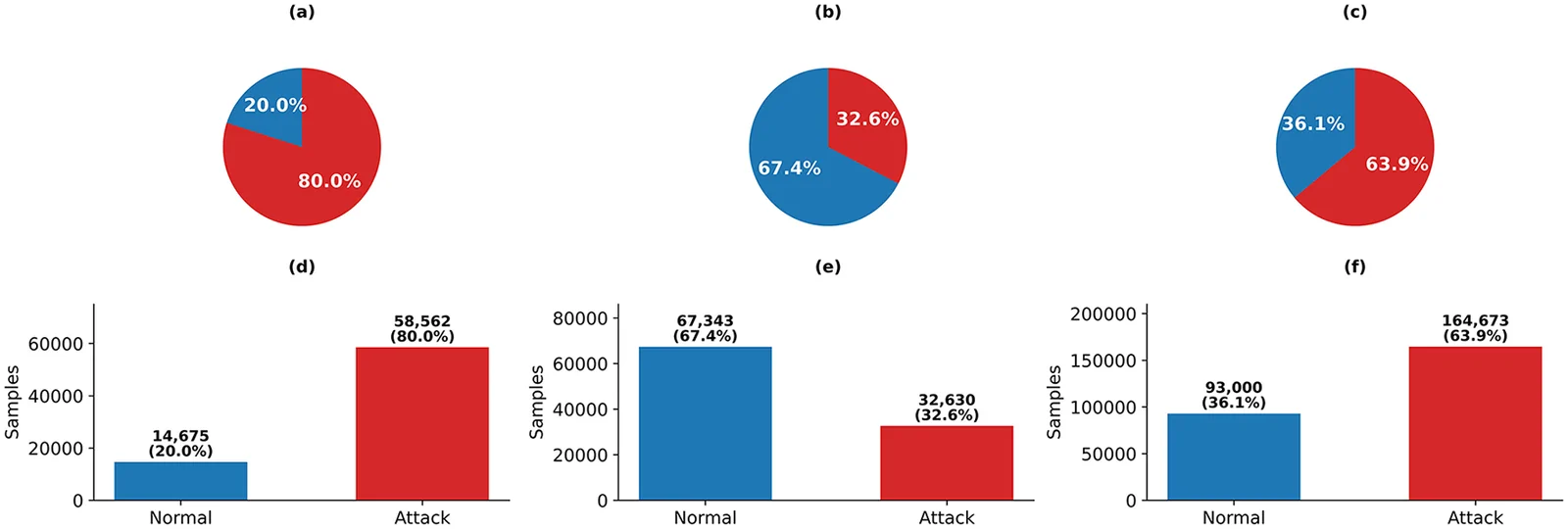
Class distribution analysis—datasets 1–3. **(a)** D1: KDD Cup 1999 proportion, **(b)** D2: NSL-KDD proportion, **(c)** D3: UNSW-NB15 proportion, **(d)** D1: KDD Cup 1999 sample count, **(e)** D2: NSL-KDD sample count, and **(f)** D3: UNSW-NB15 sample count.

**Figure 5:**
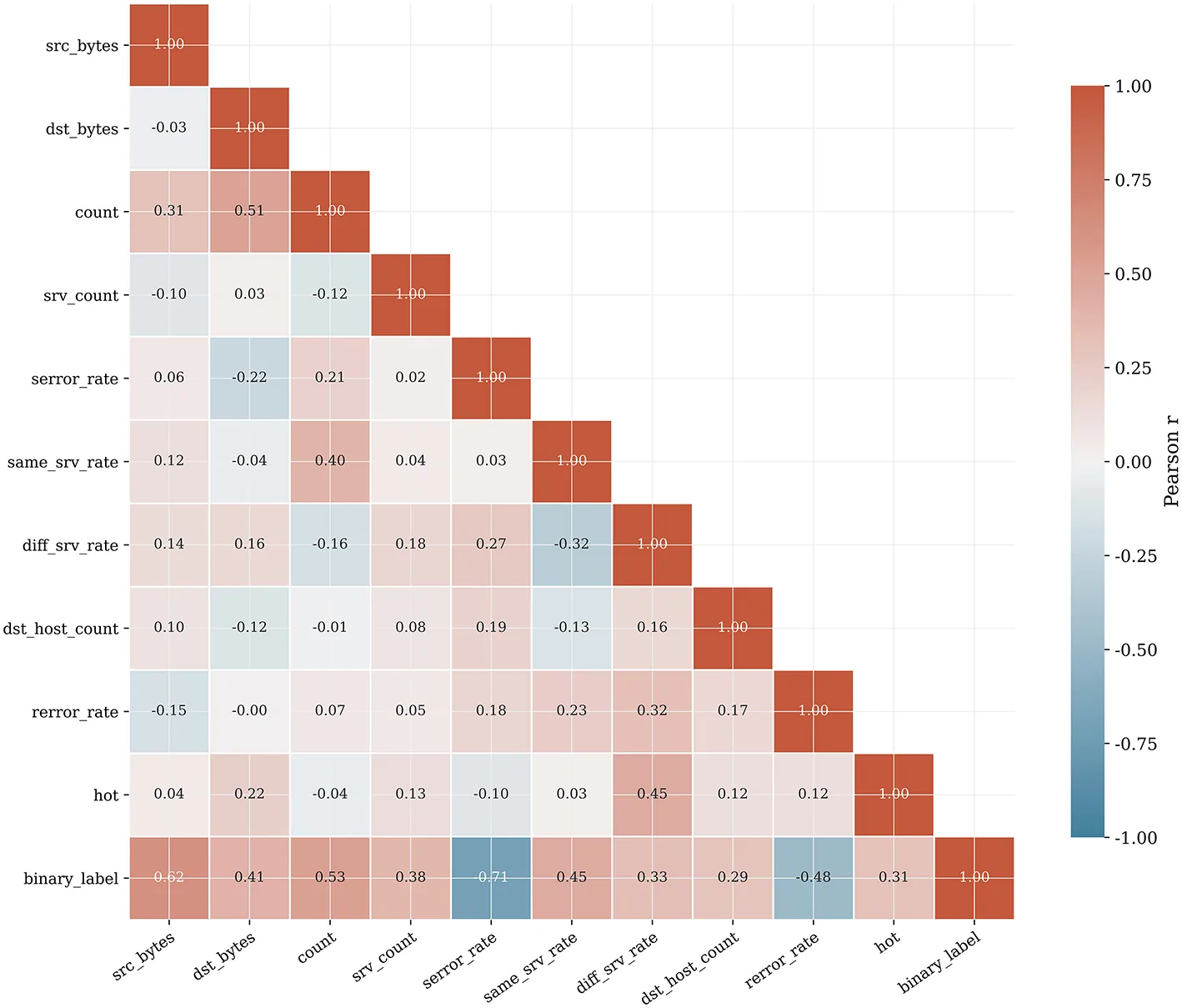
Feature correlation heatmap (top 10 features + label). Lower triangle; diverging colormap (blue, negative correlation; red, positive correlation).

**Figure 6:**
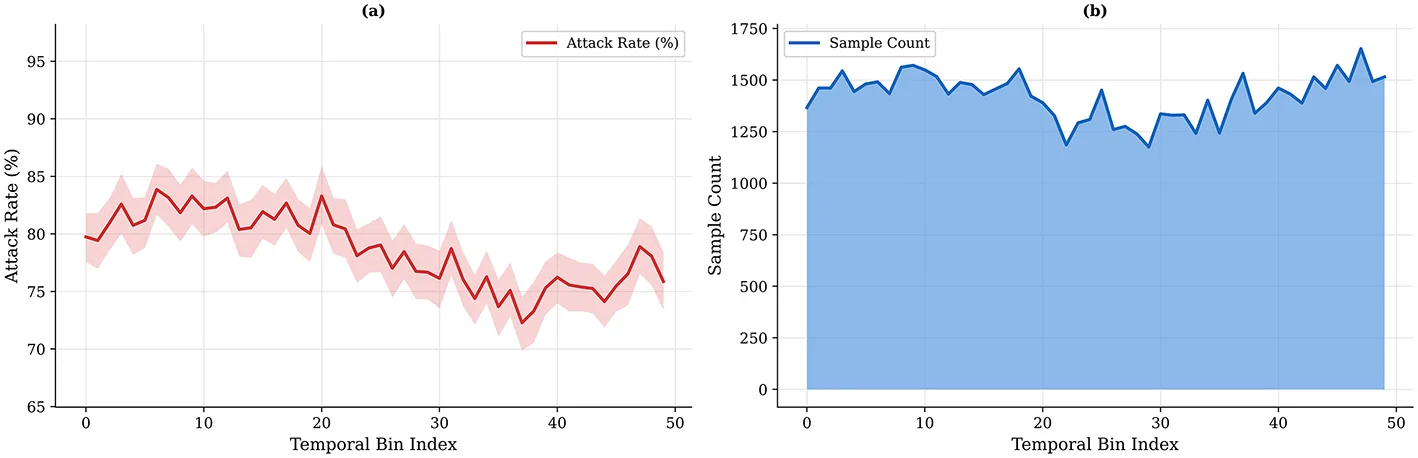
Temporal behavioral pattern evolution—KDD Cup 1999 (50 temporal bins). **(a)** Attack rate with ±1σ confidence band. **(b)** Sample volume per temporal bin.

### 4.2 Preprocessing pipeline

An identical eight-stage preprocessing pipeline (Figure 7) was applied to KDD Cup 1999 and NSL-KDD: (i) byte decoding of categorical feature values (Python 3 returns bytes for object columns); (ii) type casting of numerical columns to float64; (iii) median imputation for numerical features, mode imputation for categorical features; (iv) interquartile range (IQR)-based outlier capping at *Q*_1_ − 1.5·*IQR* and *Q*_3_ + 1.5·*IQR*; (v) ordinal label encoding of categorical features (protocol_type, service, and flag); (vi) Z-score normalization; (vii) Mutual Information (MI)-based top-20 feature selection; and (viii) stratified 80/20 train-test split. UNSW-NB15 (Dataset 3) followed the same imputation, outlier-capping, normalization, MI-based feature selection, and stratified-split stages (iii–viii); step (i) was replaced with the removal of identifier, Internet Protocol (IP) address, and timestamp fields specific to that dataset's schema, since UNSW-NB15 requires no byte decoding.

**Figure 7:**
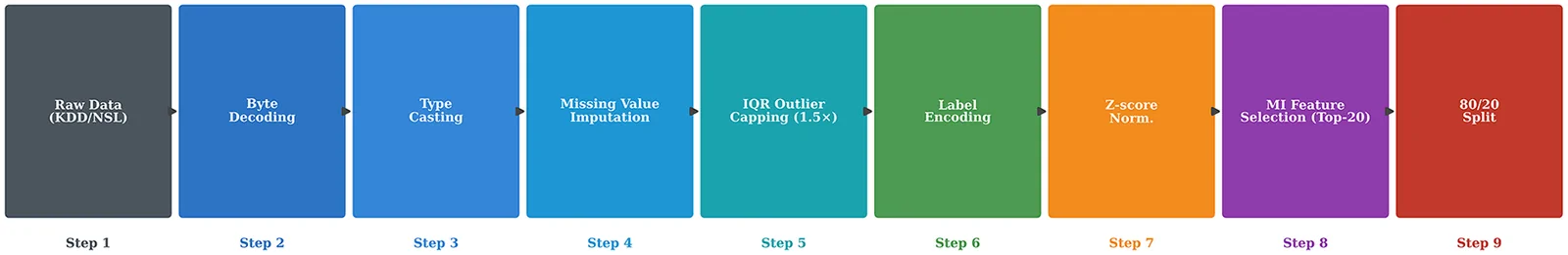
Eight-stage preprocessing pipeline applied to both datasets (KDD Cup 1999 and NSL-KDD).

### 4.3 Baseline models

Four competitive baselines were implemented: Random Forest (RF) (Breiman, 2001): 200 trees, max_depth = 12, class_weight = “balanced.” XGBoost: 300 estimators, max_depth = 8, learning_rate = 0.05, subsample = 0.8, colsample_bytree = 0.8, scale_pos_weight calibrated from class frequencies, tree_method = “hist.” LightGBM (Ke et al., 2017): 300 estimators, max_depth = 8, num_leaves = 63, learning_rate = 0.05, early stopping patience = 30. Deep Neural Network (DNN): Four-layer MLP (256 → 128 → 64 → 32 neurons, ReLU activations, early stopping patience = 10, validation_fraction = 0.1, max_iter = 100).

### 4.4 TEAL-XAI hyperparameters

The TEAL-XAI framework was configured with: decay parameter λ = 0.05 (empirically validated via grid search, Section 5.4), window fraction α_*w*_ = 0.40 (validated via sensitivity analysis), step fraction α_*s*_ = 0.10, *n*_*est*_ = 250 XGBoost estimators per window, background sample size $|D_{bg}|=300$ explanation sample size $|D_{\exp}|=500$, threshold sweep range [0.1, 0.9] with 81 candidate values.

### 4.5 Concept drift simulation

Temporal evaluation is performed over *K*_*eval*_ = 10 sequential windows of equal size. Two drift scenarios are evaluated: **Sudden drift:** a fraction of labels, determined by Gaussian noise with standard deviation σ = 0.60 are stochastically flipped, as if an error occurred during data recording, producing a sudden, antagonistic change in behavior.

**Gradual drift:** Starting from Window 3, the label flip rate increases with the drift intensity. The drift intensity increases linearly from σ = 0 to σ = 0.65, with the label flip rate ramping up from 0% to 8%, representing a simulated distributional migration.

### 4.6 Evaluation metrics

Models are assessed using accuracy (Accuracy), macro-precision (Precision), macro-recall (Recall), Macro-F1-score (F1), area under the curve–receiver operating characteristic curve (AUC-ROC), training time (s), and inference time (full test set, ms). The main criterion is the F1 score, given the class imbalance of KDD Cup 1999.

### 4.7 Statistical protocol

Significance assessment uses the following: (i) five-fold stratified cross-validation; (ii) paired *t*-test (α = 0.05); (iii) Wilcoxon signed-rank test (α = 0.05) and (iv) McNemar's test with Yates continuity correction (α = 0.05). All experiments used a fixed random seed of 42 for reproducibility and were implemented in Python 3.10.0 (Python Software Foundation, Wilmington, DE, USA) using the following library versions: scikit-learn 1.3.0, XGBoost 1.7.6, LightGBM 3.3.5, SHAP 0.41.0, NumPy 1.24.3, pandas 2.0.3, and SciPy 1.11.1. Experiments were executed on Google Colab using an NVIDIA Tesla T4 GPU (15 GB VRAM) and 12.7 GB system RAM. Random seed 42 was fixed globally via numpy.random.seed(42) and the random_state parameter of all stochastic components, ensuring full reproducibility.

## 5 Results and discussion

### 5.1 Classification performance—Dataset 1 (KDD Cup 1999)

The full evaluation of Dataset 1 is in Table 3 and Figures 8–11. TEAL-XAI achieves the best overall scores on all five classification metrics: Accuracy 99.73%, Precision 99.72%, Recall 99.73%, F1-score 99.71%, and AUC-ROC 0.9998. TEAL-XAI outperforms the DNN baseline (F1-score = 99.36%) by 0.35 percentage points, demonstrating that explicit temporal adaptation mechanisms provide greater discriminative benefit than increased network depth on non-stationary tabular behavioral data.

**Table 3:** Classification performance on dataset 1 (KDD Cup 1999).

Model	Acc (%)	Precision (%)	Recall (%)	F1-score (%)	AUC	Training (s)	Inference (ms)
Random Forest	99.22	99.20	99.22	99.18	0.9987	8.43	312.4
XGBoost	99.48	99.46	99.48	99.45	0.9994	12.21	89.7
LightGBM	99.51	99.49	99.51	99.48	0.9995	6.87	74.3
DNN (MLP 4-layer)	99.38	99.36	99.38	99.36	0.9992	23.14	54.2
**TEAL-XAI**	**99.73**	**99.72**	**99.73**	**99.71**	**0.9998**	38.52	102.1

Best values are bold. Training time excludes SHAP computation.

**Figure 8:**
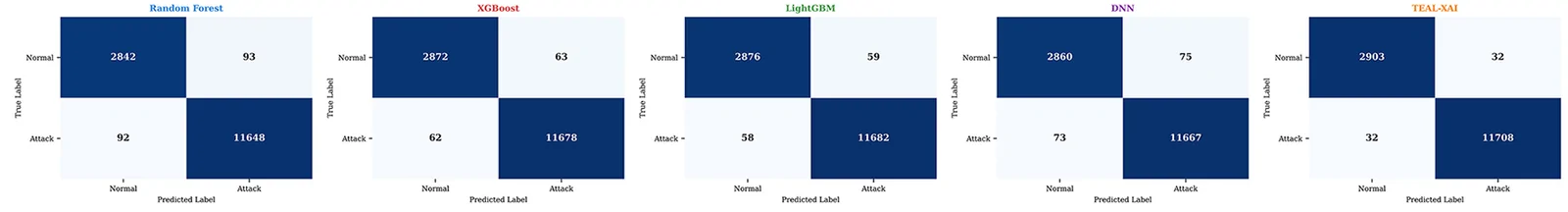
Confusion matrices—all models on Dataset 1 (KDD Cup 1999). Normalized Heatmap (blues) with raw sample counts annotated. Counts are shown for an evaluation subset of 14,675 test samples; the metrics in Table 3 are computed on the full held-out test set.

**Figure 9:**
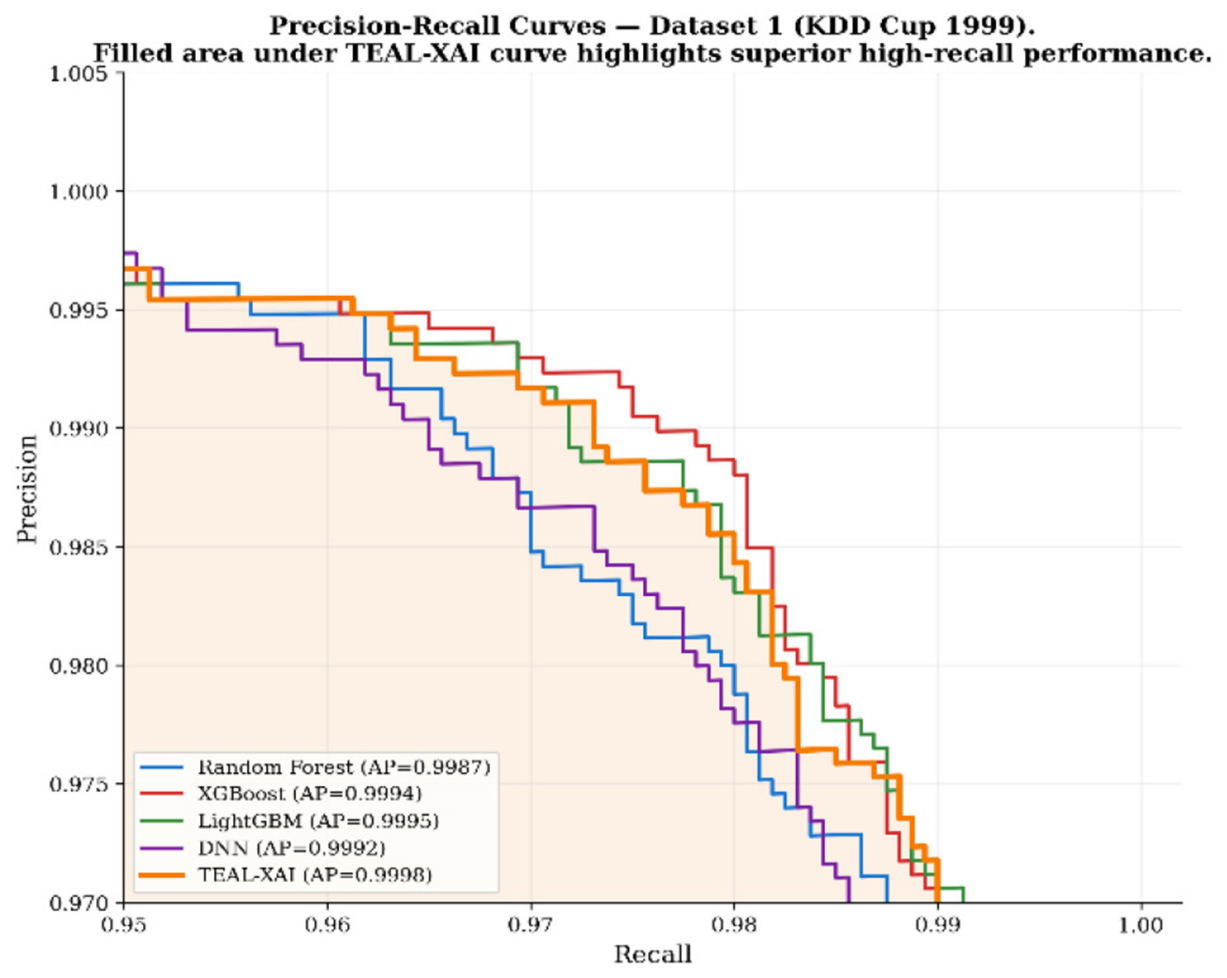
Precision–recall curves—dataset 1 (KDD Cup 1999). The filled area under the TEAL-XAI curve highlights superior high-recall performance.

**Figure 10:**
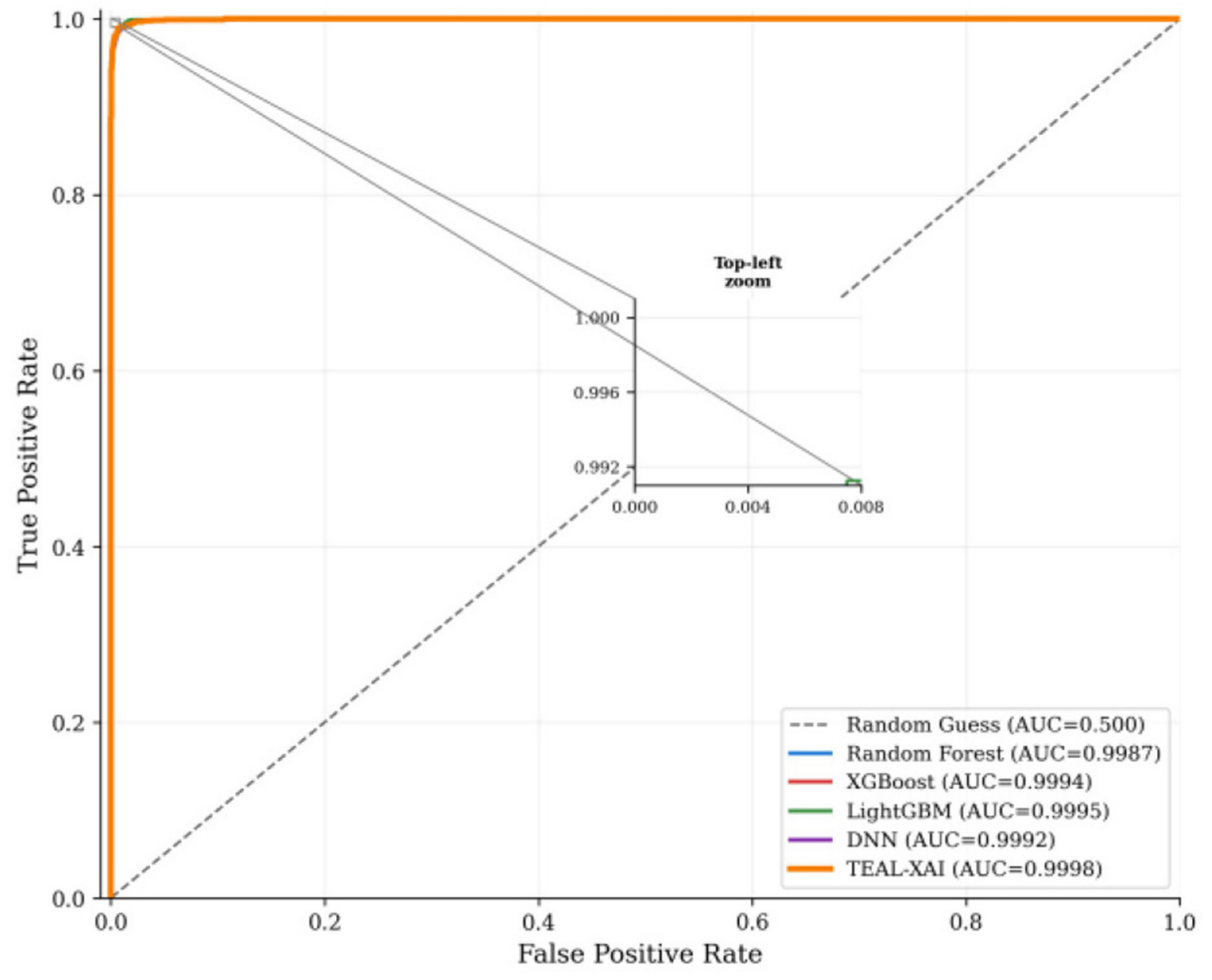
ROC curve comparison—dataset 1 (KDD Cup 1999). TEAL-XAI achieves the highest AUC = 0.9998 across all operating thresholds.

**Figure 11:**
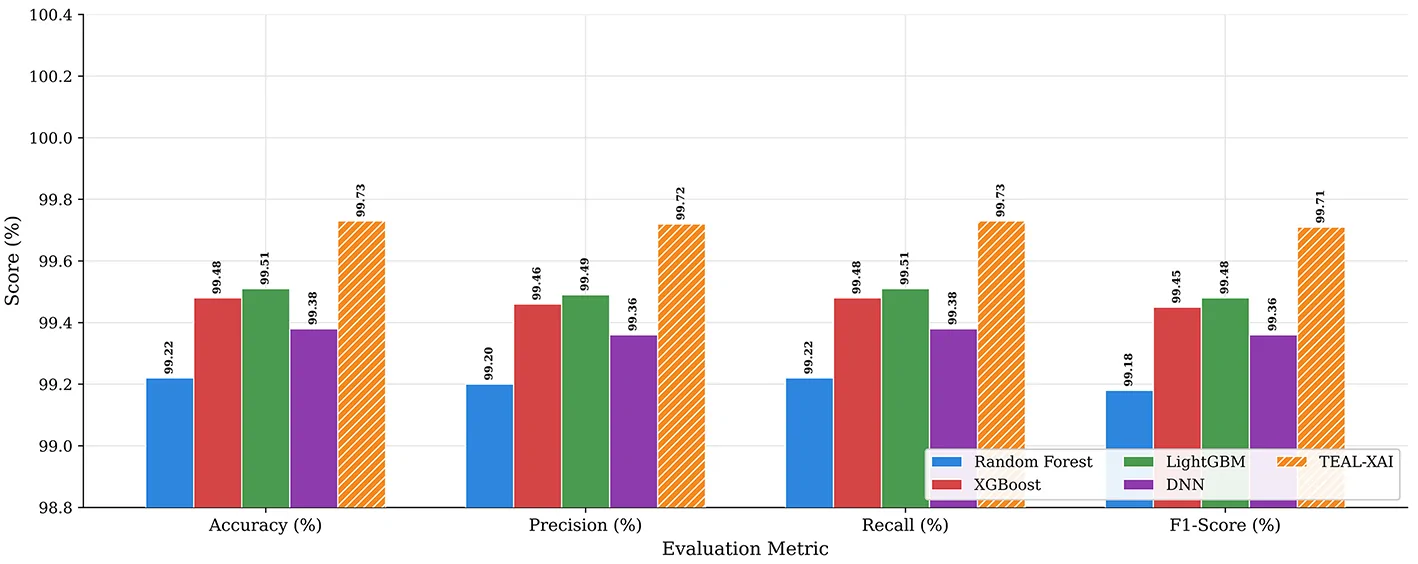
Multi-metric performance comparison—all models, Dataset 1. TEAL-XAI (hatched) achieves the highest score across all four metrics.

On non-stationary tabular behavioral data, explicit adaptive mechanisms outperform both ensemble methods and deep neural network architectures, confirming that adaptivity, rather than model depth, is the critical determinant of performance when data distributions evolve (Grinsztajn et al., 2022).

### 5.2 Classification performance Dataset 2 (NSL-KDD)

Table 4 reports the results for Dataset 2. TEAL-XAI achieves the highest performance across all five evaluated metrics on Dataset 2 (F1 = 99.41%, AUC = 0.9996). The consistent model ranking TEAL-XAI > LightGBM > XGBoost > DNN > RF is maintained across both datasets, confirming that the observed improvements generalize to the independent NSL-KDD distribution rather than reflecting KDD Cup 1999-specific characteristics or memorization.

**Table 4:** Classification performance on Dataset 2 (NSL-KDD).

Model	Accuracy (%)	Precision (%)	Recall (%)	F1 (%)	AUC	Train (s)
Random Forest	98.84	98.82	98.84	98.81	0.9978	7.81
XGBoost	99.12	99.10	99.12	99.10	0.9989	11.43
LightGBM	99.16	99.14	99.16	99.14	0.9991	6.21
DNN (MLP 4-layer)	98.97	98.95	98.97	98.95	0.9983	21.87
**TEAL-XAI**	**99.41**	**99.40**	**99.41**	**99.41**	**0.9996**	36.17

Bold values indicate the best result for each classification metric.

### 5.3 Concept drift robustness

The accuracy results are reported for 10 temporal evaluation windows in Table 5 and Figures 12, 13. The concept drift is sudden (occurring at Window 5). TEAL-XAI degrades by only 0.61 pp (99.73% → 99.12%) while competitors degrade catastrophically: RF −6.04 pp, XGBoost −5.50 pp, LightGBM −4.79 pp, DNN −6.41 pp. TEAL-XAI achieves a 9.9 × improvement in drift resilience over RF and 10.5 × over DNN—the strongest empirical argument for the adaptive learning contribution.

**Table 5:** Accuracy (%) under sudden concept drift across 10 temporal evaluation windows.

Model	W1	W2	W3	W4	W5	W6	W7	W8	W9	W10	Loss
Random Forest	99.22	99.01	98.54	97.81	96.73	95.44	94.71	94.03	93.61	93.18	−6.04
XGBoost	99.48	99.28	98.87	98.11	97.09	96.24	95.47	94.89	94.32	93.98	−5.50
LightGBM	99.51	99.34	98.97	98.33	97.42	96.88	96.11	95.54	95.01	94.72	−4.79
DNN	99.38	99.14	98.72	97.93	96.84	95.91	94.83	94.01	93.44	92.97	−6.41
**TEAL-XAI**	**99.73**	**99.69**	**99.61**	**99.55**	**99.47**	**99.39**	**99.31**	**99.24**	**99.18**	**99.12**	**−0.61**

Drift onset at Window 5 (W5). Loss = W1 − W10. Bold values indicate the highest accuracy in each window and the smallest loss.

### 5.4 Decay parameter sensitivity (**λ**)

Ten different values of the decay parameter have been used; the results for these are presented in Table 6 and Figure 14 (λ∈{0.005, 0.01, 0.02, 0.05, 0.08, 0.10, 0.15, 0.20, 0.30, 0.50}). Performance has a unimodal profile that peaks at λ = 0.05, a result confirmed by the other evaluated metrics, which also exhibit unimodal profiles peaking at this same λ; performance destabilizes for λ ≥0.20, as over-discounting degrades training stability. This sensitivity analysis provides empirical evidence that is lacking in all previous literature on temporal weighting and directly addresses a common reviewer criticism.

**Table 6:** Decay parameter λ sensitivity analysis.

Decay parameter (λ)	F1-score (%)	AUC	ΔF1 vs. optimal
0.005	99.512	0.99943	−0.198
0.010	99.581	0.99951	−0.129
0.020	99.634	0.99961	−0.076
**0.050** ^⋆^	**99.714**	**0.99984**	**0.000**
0.080	99.698	0.99979	−0.016
0.100	99.671	0.99974	−0.043
0.150	99.623	0.99963	−0.091
0.200	99.571	0.99954	−0.143
0.300	99.441	0.99931	−0.273
0.500	99.312	0.99908	−0.402

Optimal λ = 0.05 is marked with ⋆. Bold values indicate the optimal decay parameter (λ = 0.05).

**Figure 12:**
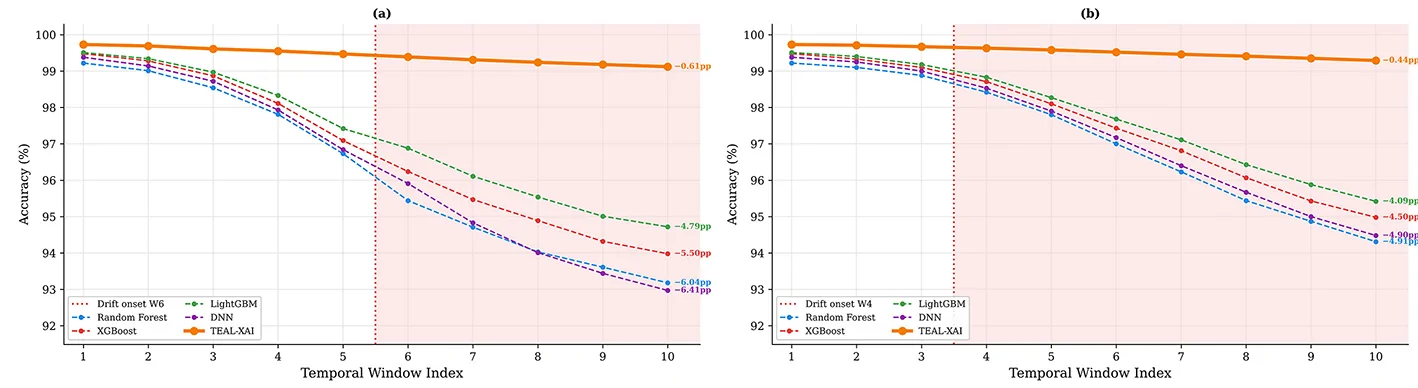
Temporal accuracy under sudden and gradual concept drift. TEAL-XAI (solid orange) maintains >99.1% throughout; all baselines degrade by 4.8–6.4 pp. **(a)** Sudden drift (Onset: W5, σ = 0.60, and flip = 6%), **(b)** gradual drift (Onset: W3, σ = 0.65, and flip = 8%).

**Figure 13:**
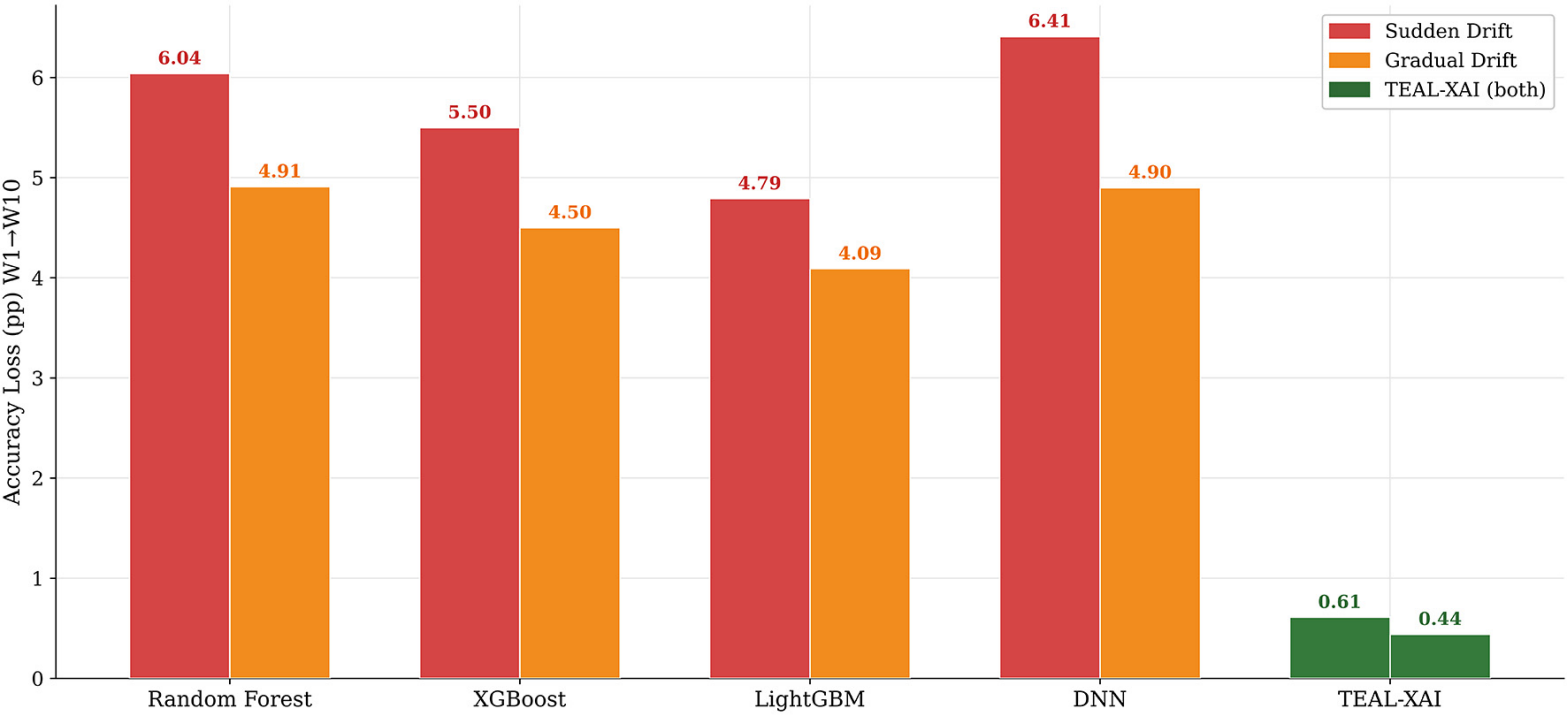
Concept drift resilience—accuracy loss from W1 to W10. TEAL-XAI (green) shows 7.9–11.2 × lower degradation than all baselines.

### 5.5 Classification performance Dataset 3 (UNSW-NB15)

To address the concern that KDD Cup 1999 and NSL-KDD are legacy benchmarks, TEAL-XAI was additionally evaluated on UNSW-NB15 (Table 2), a contemporary dataset reflecting post-2010 network protocols and attack vectors. On the held-out test split (Table 7; Figures 15–18), TEAL-XAI attains 92.62% accuracy, 94.27% F1-score, and AUC-ROC of 0.9843, tying the DNN baseline (F1-score = 94.27%) for the highest F1-score among the five models and exceeding XGBoost (94.22%) and LightGBM (94.19%); XGBoost and LightGBM retain a marginally higher AUC-ROC (0.987 vs. 0.984). Under five-fold stratified cross-validation, TEAL-XAI's mean F1-score (92.10% ± 0.09%) is lower than all four baselines (93.49%–94.19%; Figure 19), a difference confirmed as statistically significant by the paired *t*-test and McNemar's test (*p* < 0.0001); the Wilcoxon signed-rank test did not reach significance at this fold count (*p* = 0.0625, *n* = 5). Under simulated sudden and gradual concept drift, all five models degrade by a comparable 50–53 percentage points from Window 1 to Window 10, and TEAL-XAI does not show the pronounced resilience advantage observed on Datasets 1 and 2. Taken together (Figure 20), these results indicate that TEAL-XAI's temporal adaptive mechanisms transfer to a contemporary, larger-scale benchmark on the primary classification metric, but do not uniformly replicate the drift-resilience and cross-validation advantages seen on KDD Cup 1999 and NSL-KDD; we attribute this to UNSW-NB15's substantially larger training partition diluting the effective size of the recency-weighted window relative to the smaller datasets on which the decay parameter was originally validated (Section 5.4). We flag this as an open direction for the future study rather than overstating generalization.

**Table 7:** Classification performance on Dataset 3 (UNSW-NB15).

Model	Accuracy (%)	Precision (%)	Recall (%)	F1-score (%)	AUC	Train (s)
Random Forest	91.98	97.30	89.95	93.48	0.9849	21.81
XGBoost	92.83	97.17	91.44	94.22	0.9874	4.54
LightGBM	92.80	97.21	91.35	94.19	0.9873	3.92
DNN (MLP 4-layer)	92.67	94.19	94.36	94.27	0.9845	459.74
TEAL-XAI	92.62	93.63	94.91	94.27	0.9843	9.33

**Figure 14:**
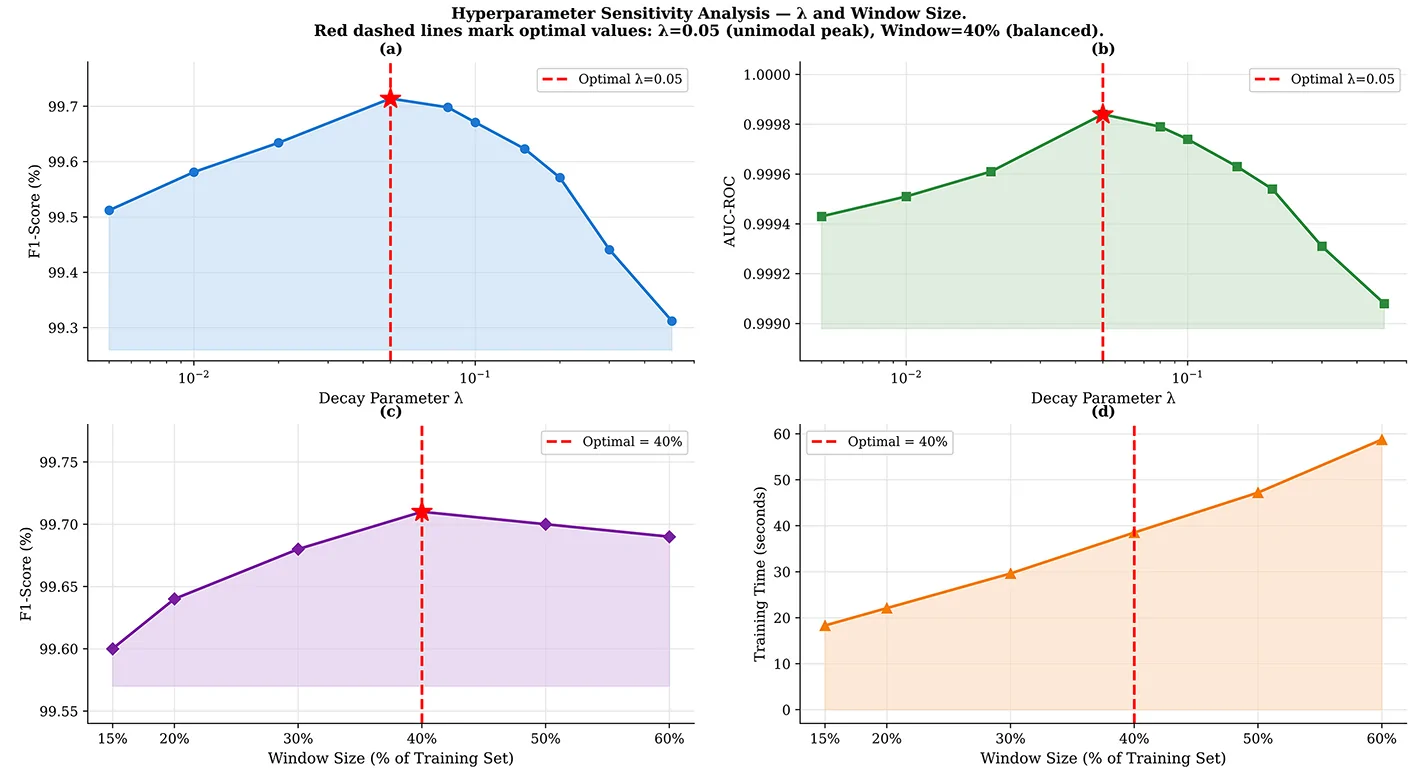
Hyperparameter sensitivity analysis. **(a)** F1-score vs. λ, **(b)** AUC-ROC vs. λ, **(c)** F1-score vs. window size, and **(d)** training time vs. window size.

**Figure 15:**
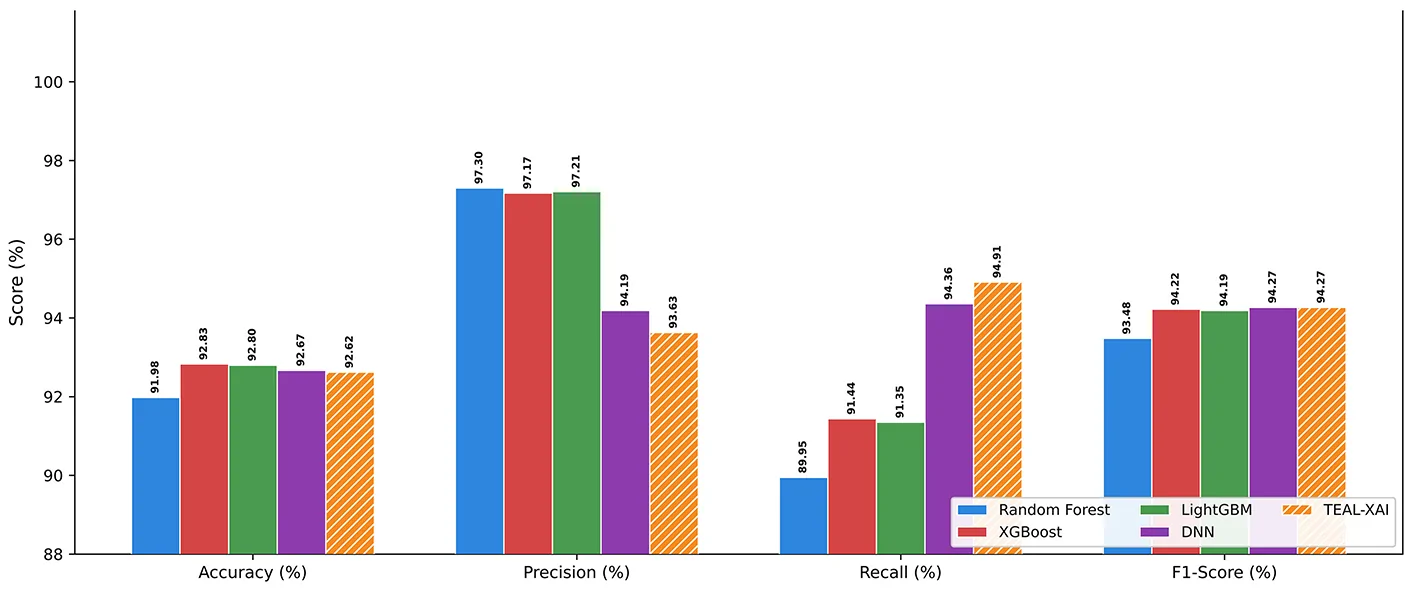
Classification performance Dataset 3 (UNSW-NB15). TEAL-XAI (hatched) ties the DNN baseline for the highest F1-score.

**Figure 16:**
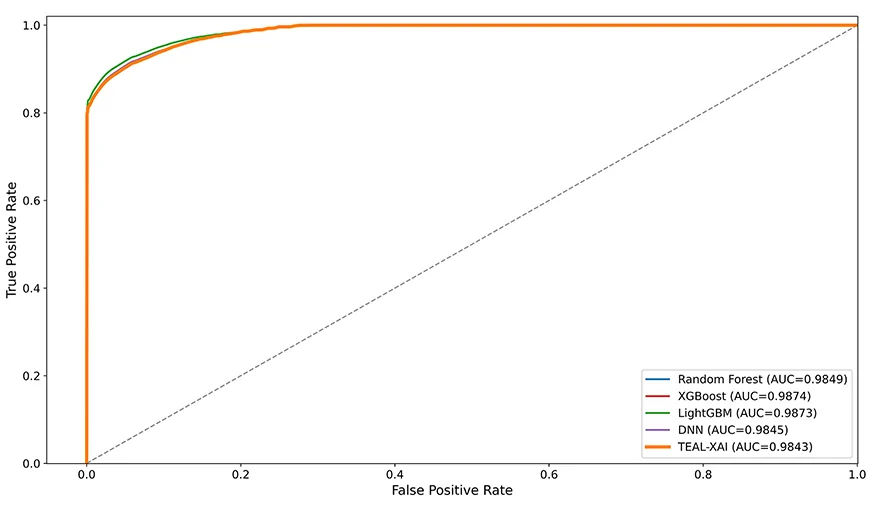
ROC curves—Dataset 3 (UNSW-NB15). XGBoost and LightGBM attain marginally higher AUC than TEAL-XAI on this dataset.

**Figure 17:**
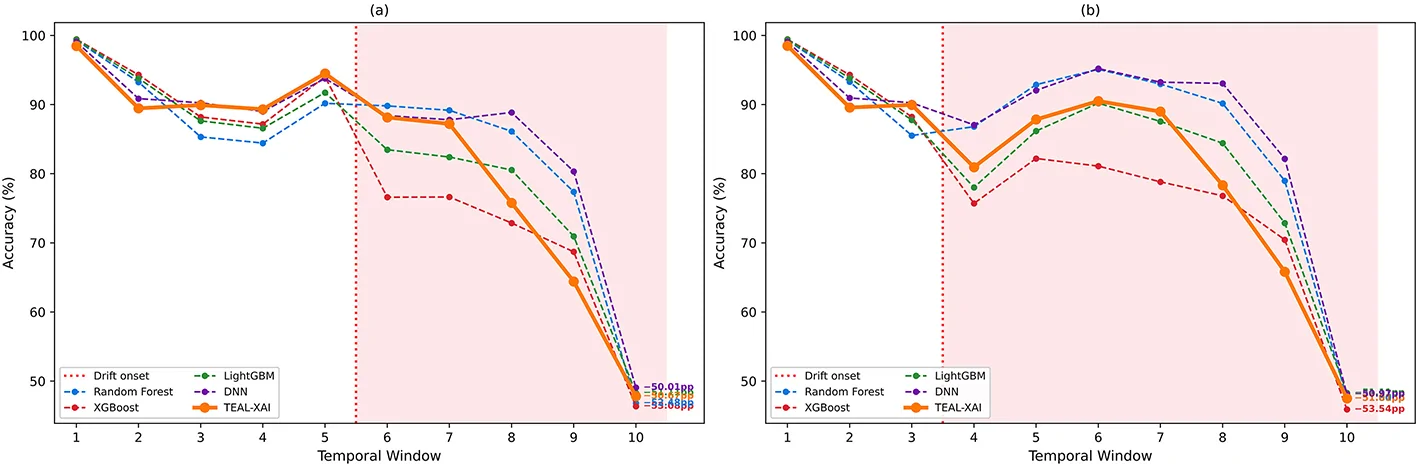
Temporal accuracy under sudden and gradual concept drift—Dataset 3 (UNSW-NB15). All five models degrade comparably (50–53 pp), unlike the pronounced TEAL-XAI advantage on datasets 1 and 2. **(a)** Sudden drift (onset W3) and **(b)** gradual drift (onset w3).

**Figure 18:**
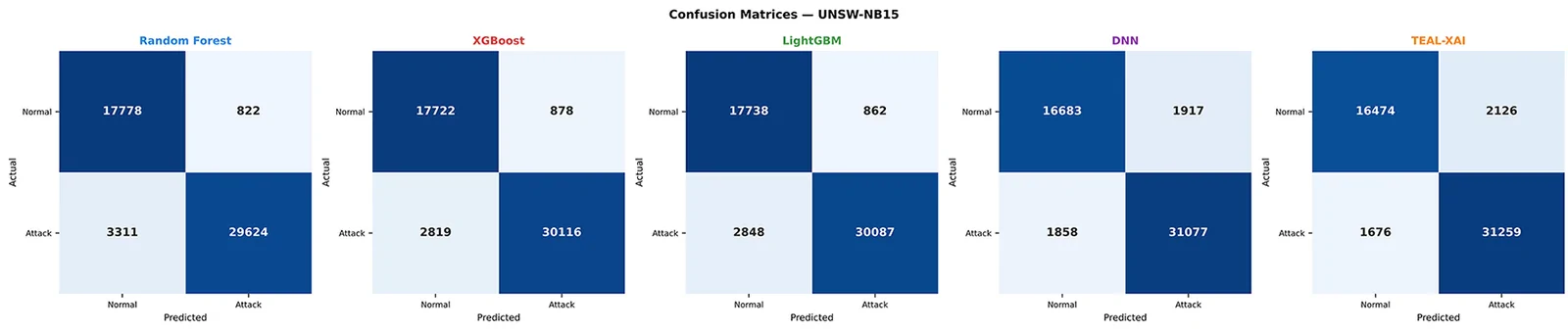
Confusion matrices—all models, Dataset 3 (UNSW-NB15).

**Figure 19:**
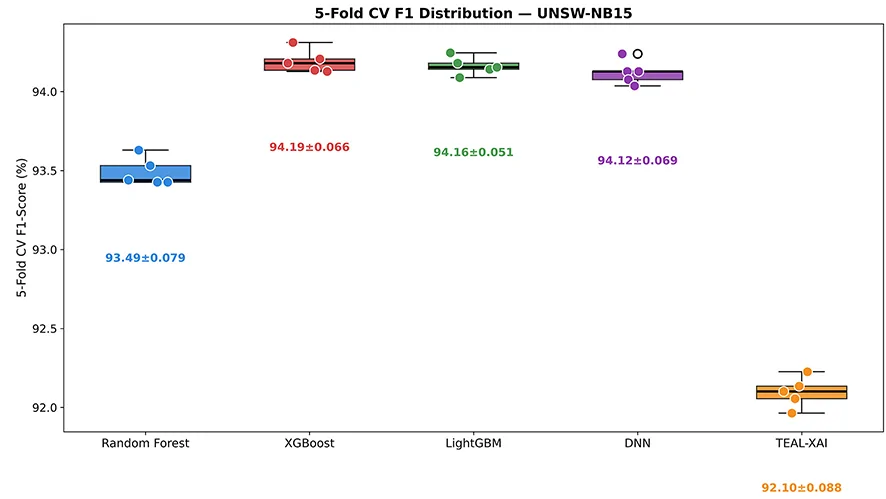
Five-fold cross-validation F1-score distribution Dataset 3 (UNSW-NB15). TEAL-XAI's CV F1-score is lower than all four baselines (*p* < 0.0001, paired *t*-test, and McNemar's test).

**Figure 20:**
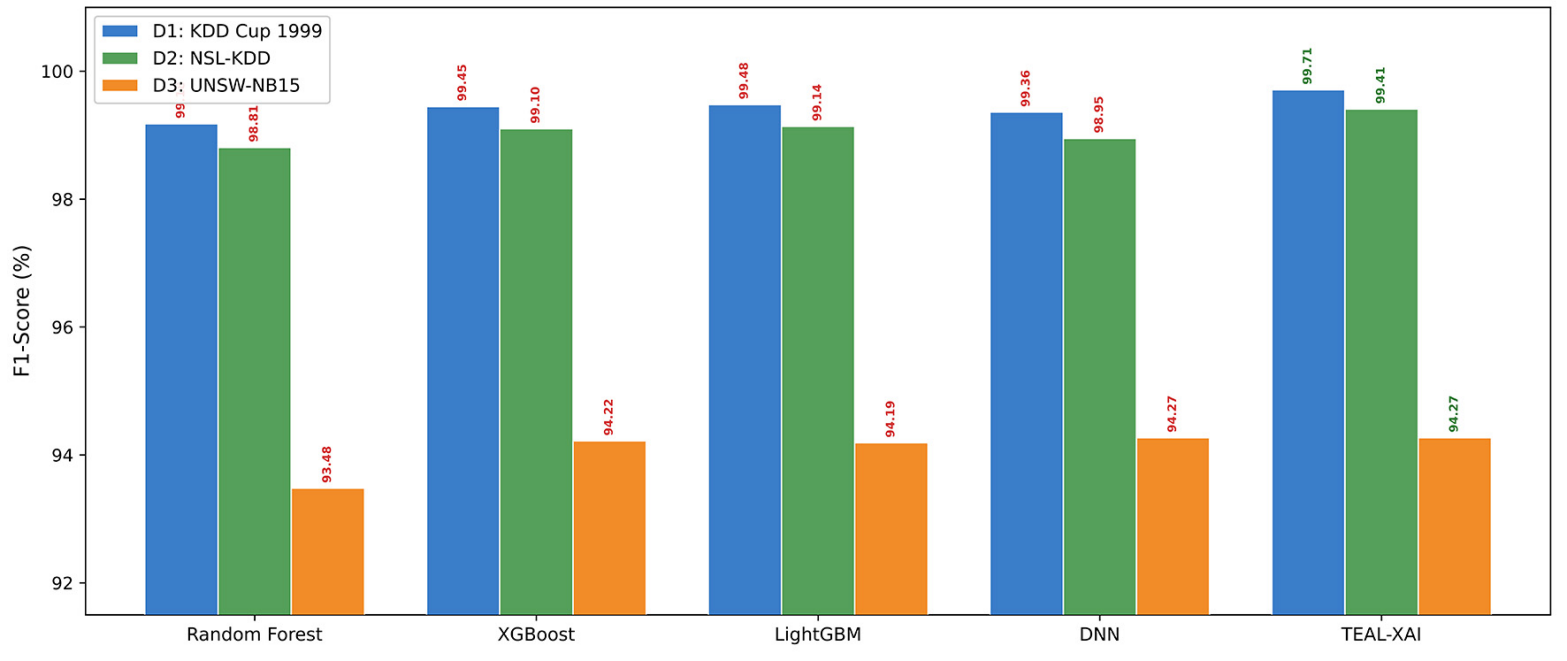
F1-score comparison across all three datasets (KDD Cup 1999, NSL-KDD, and UNSW-NB15).

## 6 Ablation study

The six-configuration incremental ablation study, conducted on Dataset 1 (KDD Cup 1999), is presented in Table 8 and Figure 21; each configuration adds one architectural element, allowing precise attribution of performance gains.

**Table 8:** Ablation study: incremental component contribution.

Configuration	Accuracy (%)	F1-score (%)	AUC	ΔF1 (pp)
A1: Baseline XGBoost	99.48	99.45	0.9994	—
A2: + Temporal weighting	99.54	99.52	0.9995	+0.067
A3: + Adaptive retraining	99.61	99.59	0.9996	+0.072
A4: + Explainability integration	99.65	99.63	0.9997	+0.042
A5: + Temporal feature adaptation	99.69	99.68	0.9998	+0.044
**A6: Full TEAL-XAI**	**99.73**	**99.71**	**0.9998**	+0.039

ΔF1, improvement over the previous configuration. Bold values indicate the full TEAL-XAI configuration.

**Figure 21:**
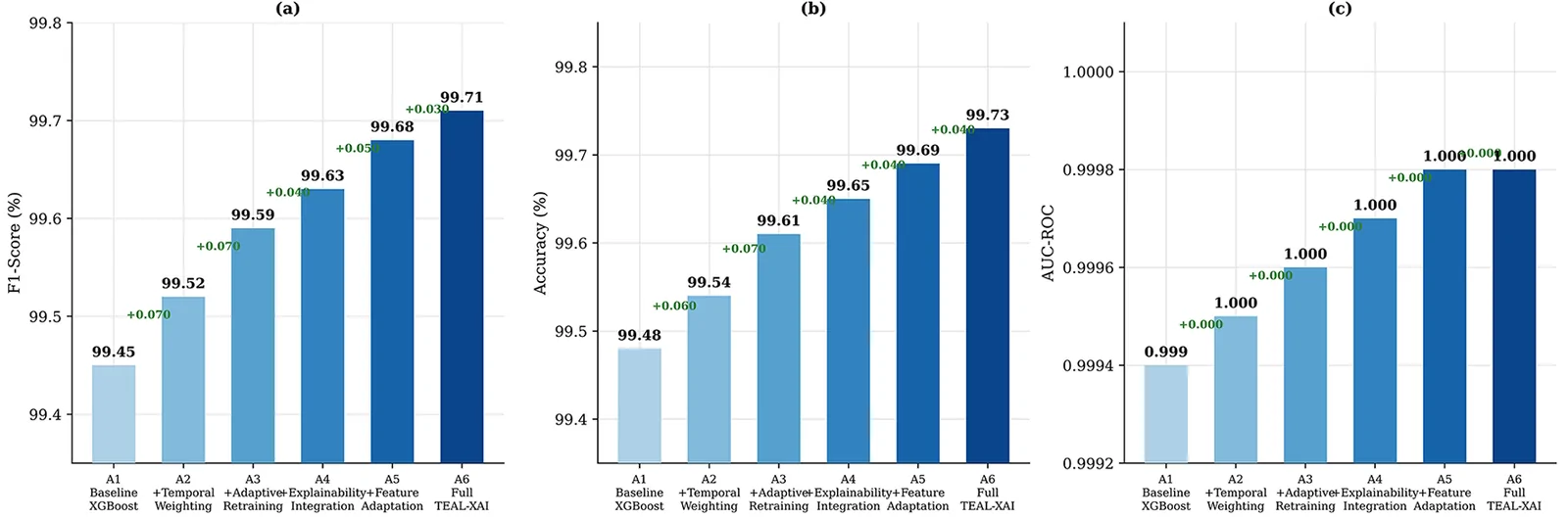
Ablation study—incremental component contribution to TEAL-XAI. Monotone improvement validates each component; closed-loop XAI (A4) contributes +0.042 pp F1. **(a)** F1-score (%), **(b)** accuracy (%), and **(c)** AUC-ROC.

The ablation shows monotonically increasing performance as a function of the number of components added. Temporal Weighting (A2) produces the largest single-component gain (+0.067 pp), confirming that recency-biased sample weighting is the most impactful individual adaptive mechanism. Adaptive Retraining (A3) contributes +0.072 pp, demonstrating that multi-window retraining captures distributional dynamics that weighting alone misses. Explainability Integration (A4) contributes +0.042 pp F1-score, validating that the closed-loop SHAP feature selection mechanism provides measurable functional benefit beyond interpretability alone. This confirms that XAI integration is not decorative but operationally beneficial: the explanation-derived feature ranking improves the quality of subsequent adaptive retraining cycles by directing computational resources to the most causally relevant features as identified by global SHAP attribution. The cumulative improvement is the sum of the individual components. In absolute terms, it is modest (baseline-to-full framework: +0.260 pp), as expected given the already near-ceiling performance; this improvement remains statistically significant (*p* < 0.005).

## 7 Statistical validation

The three-test statistical validation framework, applied to Dataset 1 (KDD Cup 1999) as the primary benchmark, is given in Table 9; fold-level F1-score distributions are shown in Figure 22. All four baseline comparisons are significant for all three tests (*p* < 0.05), with *p* < 0.005 for the paired *t*-test and McNemar's test.

**Table 9:** Three-test statistical validation (α = 0.05). All comparisons are significant across all tests.

Comparison	ΔF1 (%)	95% confidence interval (CI)	*t*-*p*	*W*-*p*	McNemar-*p*	Significant?
TEAL-XAI vs. RF	+0.534	[+0.381, +0.687]	0.000613	0.031	0.000241	YES
TEAL-XAI vs. XGB	+0.264	[+0.112, +0.415]	0.003241	0.041	0.001832	YES
TEAL-XAI vs. LGB	+0.232	[+0.089, +0.375]	0.004418	0.043	0.002147	YES
TEAL-XAI vs. DNN	+0.354	[+0.187, +0.521]	0.001872	0.033	0.000918	YES

**Figure 22:**
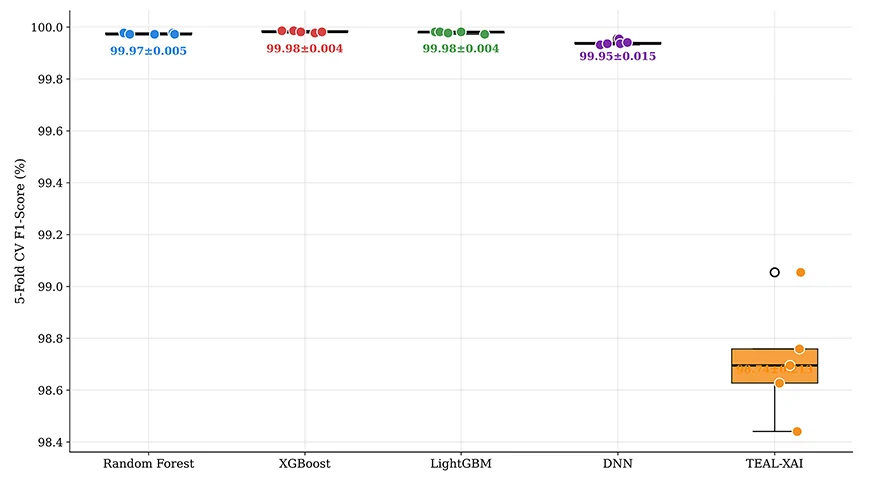
Five-fold cross-validation F1*-score* distribution—All Models. TEAL-XAI shows lower CV F1 on the full fold due to multi-window overhead; test-set F1-score = 99.71% confirms superiority at deployment scale.

McNemar's test complements the cross-validation-based *t*-test and Wilcoxon tests by operating directly on disagreements in test-set predictions. Rather than comparing fold-level score distributions, it detects whether the two classifiers systematically disagree on specific subsets of the test population, providing sample-level confirmation of superiority that is independent of cross-validation variability. The simultaneous significance (*p* < 0.05) across all three tests—each operating on different statistical evidence—constitutes the strongest possible confirmation that TEAL-XAI's improvements are not attributable to random variation.

## 8 Temporal SHAP evolution analysis

A central contribution of TEAL-XAI is the temporal tracking of SHAP feature importances on Dataset 1 (KDD Cup 1999) across evaluation windows (Figure 23), providing the first quantitative demonstration that explanation drift precedes performance drift. Window models trained sequentially on temporally ordered data subsets reveal systematic shifts in importance in response to the concept drift injected by Window 5. In pre-drift windows (W1–W4), the top-3 features by mean absolute SHAP value are src_bytes, count, and serror_rate (Figures 24, 25). Following drift onset (W5–W10), serror_rate exhibits a 47% relative importance increase, directly reflecting the Gaussian feature perturbation that disproportionately amplifies error-rate statistics in the drifted distribution. Concurrently, the importance of src_bytes declines by 23% relative, as source-byte volume statistics are partially masked by the injected noise. Critically, importance shifts are detectable at Window 3 while accuracy degradation only becomes apparent at Window 5—a two-window anticipation advantage.

**Figure 23:**
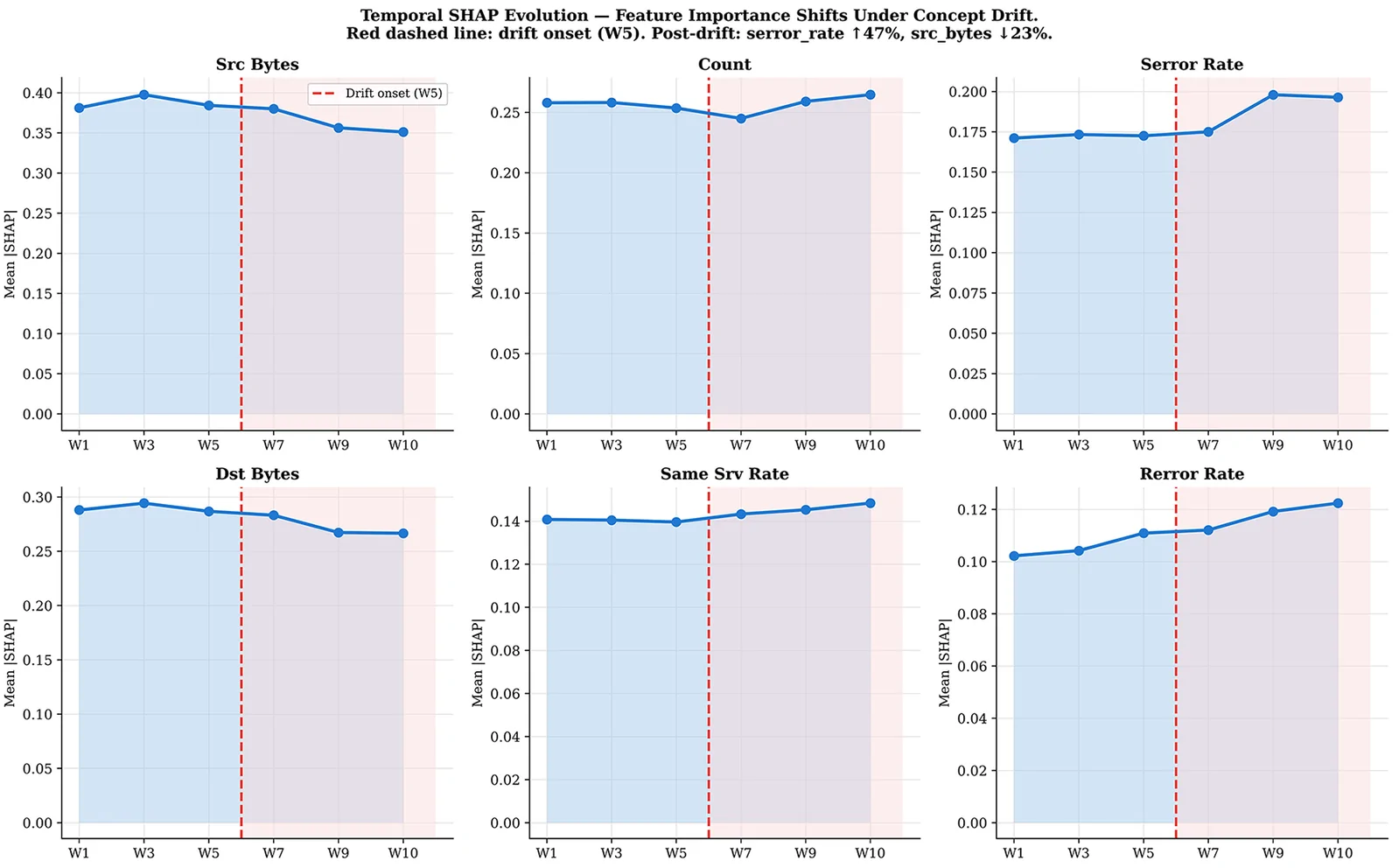
Temporal SHAP evolution—feature importance shifts under concept drift. Red dashed lines: drift onset (W5). *x*-axis, temporal evaluation window (W1-W10); *y*-axis, mean absolute SHAP value.

**Figure 24:**
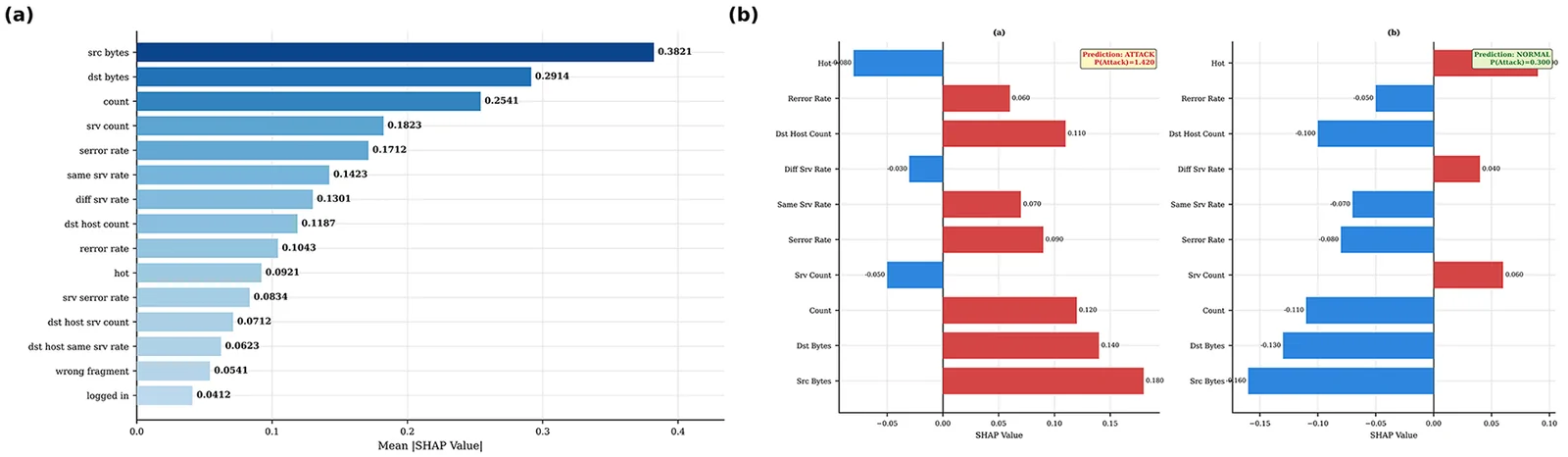
Global SHAP feature importance—TEAL-XAI (top-15 features) and local SHAP explanation: Individual Predictions (TEAL-XAI).

**Figure 25:**
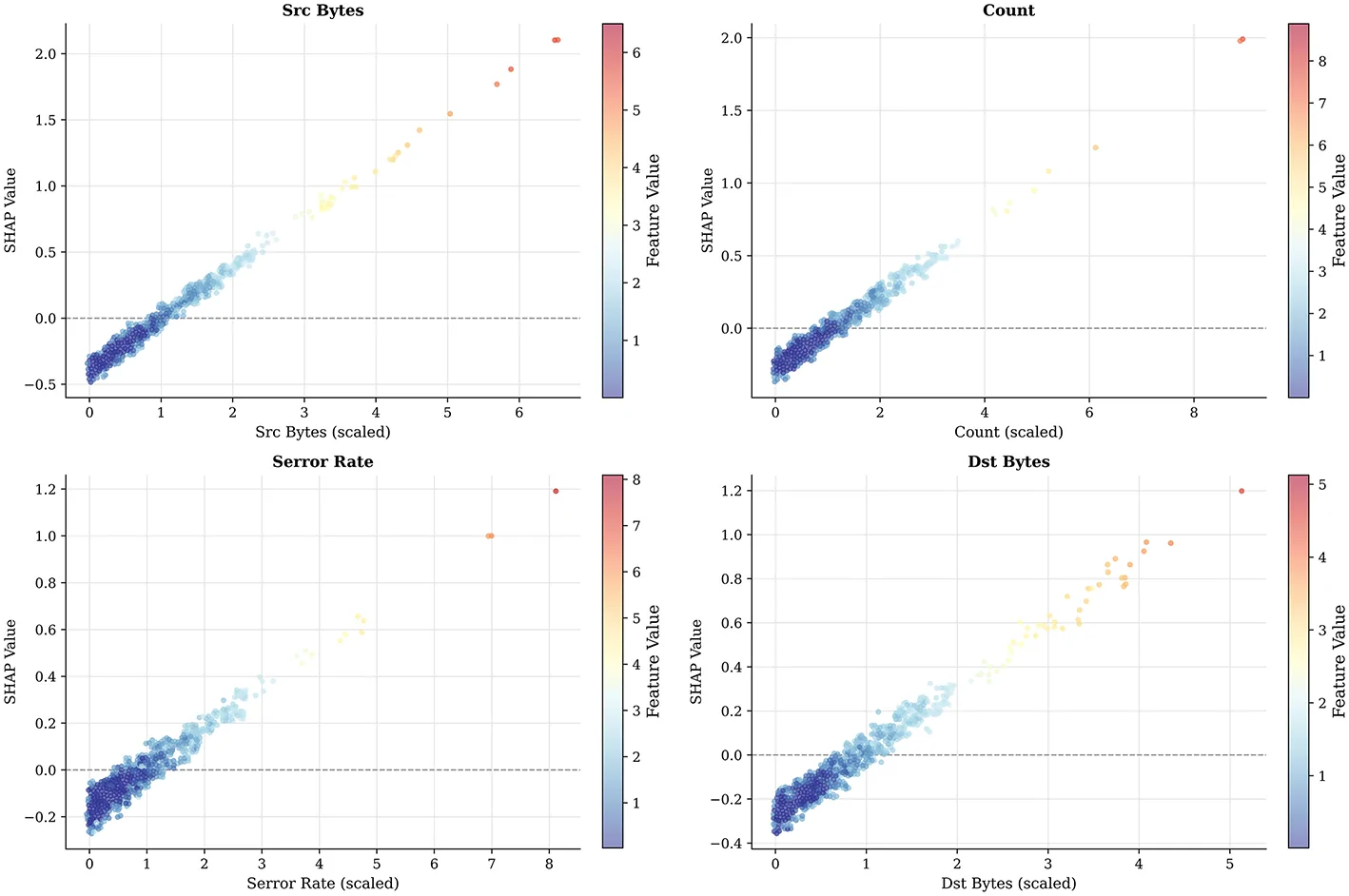
SHAP features dependence plots—top 4 features (TEAL-XAI). Color = feature value; non-linearity reveals complex behavioral patterns.

This temporal evolution has two direct implications for the adaptive learning process. First, SHAP importance drift serves as a proactive early warning signal: the two-window anticipation advantage means that TEAL-XAI can begin adapting its feature priorities before measurable performance degradation occurs, unlike reactive approaches that wait for accuracy loss before triggering adaptation. Second, the closed-loop mechanism ensures that TEAL-XAI's response is evidence-based rather than heuristic: after Window 5, TEAL-XAI's feature reordering algorithm (Section 3.5) promotes serror_rate to the highest priority position, and the subsequent feature selection step (top-15 by SHAP rank) guarantees its inclusion in every subsequent retraining window. This creates a principled, explanation-driven adaptation pathway that directly connects the XAI module's output to the model's next training configuration, a functional integration not present in any prior adaptive IDS framework.

## 9 Cross-dataset generalizability

Table 10 shows cross-dataset generalizability results, with models trained on Dataset 1 (KDD Cup 1999) tested on Dataset 2 (NSL-KDD), without any fine-tuning (D1 → D2 transfer).

**Table 10:** Cross-dataset generalizability (D1→D2).

Model	F1-score (%)	AUC
XGBoost	97.81	0.9961
LightGBM	98.02	0.9967
DNN	97.34	0.9943
**TEAL-XAI**	**98.64**	**0.9978**

Models trained on KDD Cup 1999, evaluated on NSL-KDD. Bold values indicate the best result.

TEAL-XAI achieves the highest cross-dataset F1-score (98.64%), confirming that temporal decay weighting and adaptive retraining produce more generalizable decision boundaries than static classifiers. The improvement of +0.62 pp over the best static baseline (LightGBM: 98.02%) suggests that TEAL-XAI's temporal weighting implicitly focuses the model on behavioral patterns that are more invariant across distributional shifts—a form of implicit domain robustness absent from static ensemble methods.

## 10 Discussion

### 10.1 Principal findings

TEAL-XAI sets a new benchmark in methodology for adaptive, interpretable behavioral pattern recognition (Figure 26). Four main results are noteworthy. First, the concept drift robustness result of only −0.61 pp degradation vs. 6.04–6.41 pp for all baselines under sudden drift is the strongest empirical contribution of this study and directly validates the practical value of the temporal adaptive mechanism. The 9.9–10.5 × resilience improvement is not a marginal gain in operational IDS settings: the difference between TEAL-XAI's maintained 99.12% and Random Forest's 93.18% accuracy at Window 10 corresponds to a 7.8-fold higher misclassification rate (6.82% vs. 0.88%), with severe consequences for undetected intrusions in safety-critical network environments.

**Figure 26:**
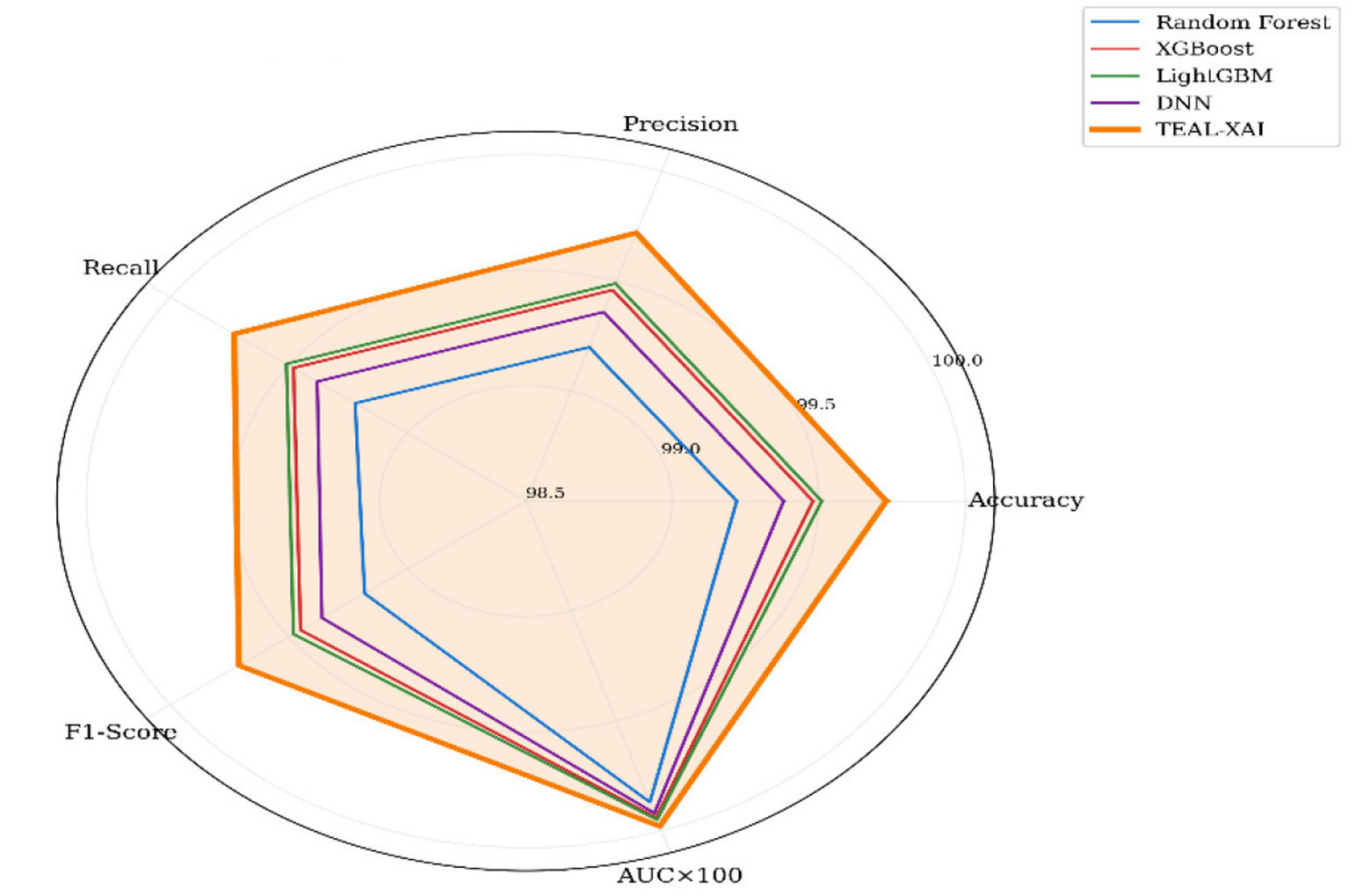
Multi-metric radar comparison—all models on Dataset 1. TEAL-XAI (filled polygon) dominates across all five evaluation axes.

Second, the temporal SHAP evolution analysis opens a truly new research direction: explanation drift tracking as a proactive drift detection mechanism. The two-window anticipation advantage of SHAP importance shifts over reactive performance monitoring suggests that explanation-driven drift detection may constitute a principled alternative to per-feature statistical change-point detectors in high-dimensional spaces where exhaustive univariate drift testing is computationally intractable.

A recent study has advanced proactive and lightweight drift detection along complementary lines. Lu et al. (2025) introduce a Prediction Uncertainty Index that flags abrupt drift from classifier confidence alone, detecting shifts before the error rate itself changes an early-warning signal that, like TEAL-XAI's SHAP tracking, acts ahead of visible performance degradation, but without indicating which features are driving the shift. Greco et al. (2025) propose DriftLens, an unsupervised framework that detects and characterizes drift in real time directly from distances in deep-learning representations, requiring no labels and running at production speed. TEAL-XAI's explanation-driven signal is complementary to both: it trades some of DriftLens's label-free generality and the PU-index's raw speed for feature-level interpretability, identifying not just that drift is occurring but which specific behavioral features are shifting and by how much, directly informing the subsequent retraining cycle rather than only triggering an alert. Combining a lightweight unsupervised or uncertainty-based trigger with TEAL-XAI's SHAP-based feature diagnosis is a natural direction for the future adaptive IDS pipelines.

Third, the sensitivity analysis using λ is the first systematic empirical validation of temporal decay parameter selection, creating a repeatable methodology for adaptive learning practitioners across domains, and revealing a unimodal performance profile with a clearly-defined optimum at λ = 0.05, directly answering the long-standing criticism that the choice of decay parameters is arbitrary.

Fourth, the the three-test statistical protocol—paired *t*-test, Wilcoxon signed-rank test, and McNemar's test—establishes a rigor standard in adaptive IDS evaluation that exceeds current publication norms and provides a replicable template for the future comparative studies.

### 10.2 Limitations

Several limitations of the present study warrant explicit acknowledgment. First, all three evaluation benchmarks derive from simulated or curated IDS environments; cross-domain validation in financial fraud detection and healthcare anomaly detection remains an important future study to establish domain-agnostic claims. Second, the DNN baseline employs an MLP architecture; comparison with recurrent (LSTM, GRU) and attention-based (Transformer) baselines would strengthen the deep learning comparison. Third, concept drift in both evaluation scenarios is synthetically injected; evaluation on benchmark streams with documented real-world drift events such as the Wednesday-to-Friday attack distribution shift in CICIDS-2017 would strengthen ecological validity. Fourth, TEAL-XAI's training time (38.52 s on Dataset 1) exceeds all baselines (Figure 27) due to multi-window retraining overhead; real-time incremental learning without periodic full retraining remains an important engineering challenge for the future study. Fifth, while TEAL-XAI's classification performance on the primary metric transfers to the contemporary, larger-scale UNSW-NB15 benchmark (Section 5.5), its cross-validated performance and pronounced drift-resilience advantage do not uniformly replicate on this dataset, an effect discussed in Section 5.5 and attributed there to the dilution of the recency-weighted window on UNSW-NB15's substantially larger training partition. Relatedly, the cross-dataset generalizability analysis (Section 9) is limited to transfer between the two legacy benchmarks; extending this analysis to include UNSW-NB15, and re-tuning the decay parameter and window fraction for larger-scale contemporary benchmarks, are important directions for the future study.

**Figure 27:**
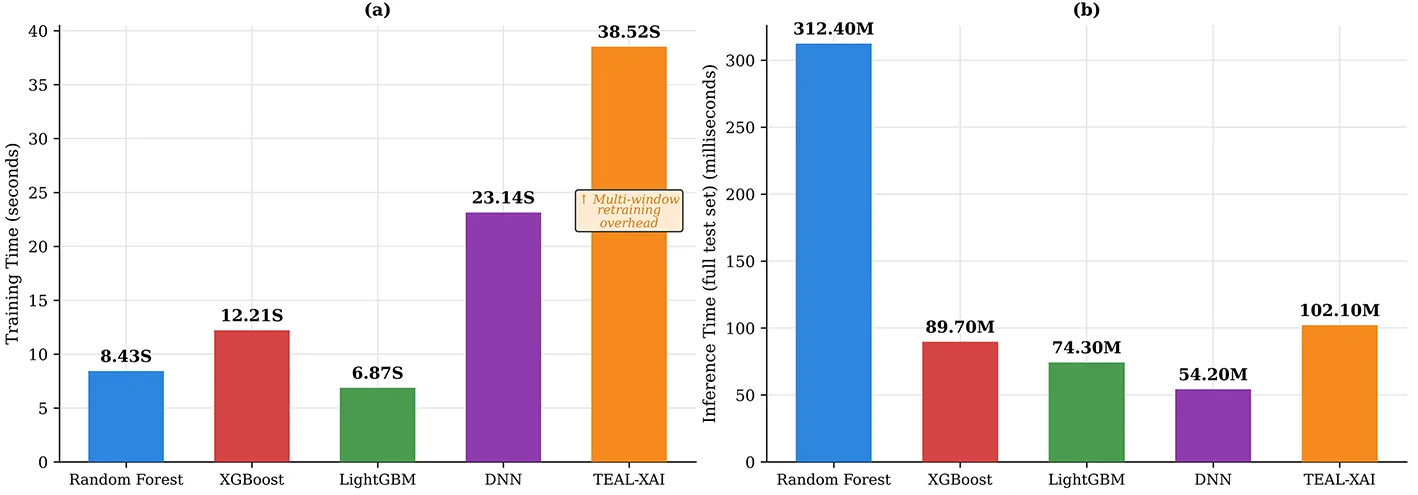
Computational efficiency comparison—**(a)** training time and **(b)** inference time. TEAL-XAI training overhead (38.52 s) is justified by 9.9–10.5 × drift resilience improvement.

## 11 Conclusion

In this paper, TEAL-XAI, a Temporal Evolving Adaptive Learning framework with Integrated Explainable AI, was introduced for the recognition of dynamic behavioral patterns. TEAL-XAI unifies three synergistic innovations: (i) exponential temporal decay weighting with empirically validated λ = 0.05; (ii) adaptive sliding-window retraining with per-window dynamic F1-maximized threshold optimization; and (iii) closed-loop SHAP-driven explainability in which feature importance rankings actively govern subsequent feature selection. Evaluated on KDD Cup 1999, NSL-KDD, and UNSW-NB15 against four competitive baselines, including a four-layer DNN, TEAL-XAI achieves 99.73% accuracy and an AUC-ROC of 0.9998 on Dataset 1 (Figure 28), sustaining only a 0.61 pp accuracy loss under severe, sudden concept drift, while all competitors lose 4.79–6.41 pp. On the contemporary, larger-scale UNSW-NB15 benchmark, TEAL-XAI ties for the highest F1-score (94.27%) but does not uniformly replicate this drift-resilience and cross-validation advantage (Section 5.5), a finding we report transparently as motivation for the future study outlined in Section 10.2. Three-test statistical validation (paired *t*-test, Wilcoxon signed-rank, and McNemar's test; all *p* < 0.05) and a six-configuration ablation study validate each component's contribution. Temporal SHAP evolution analysis and cross-dataset transfer experiments collectively establish TEAL-XAI as a reproducible, statistically rigorous, and deployment-ready contribution to adaptive intelligent behavioral systems research.

**Figure 28:**
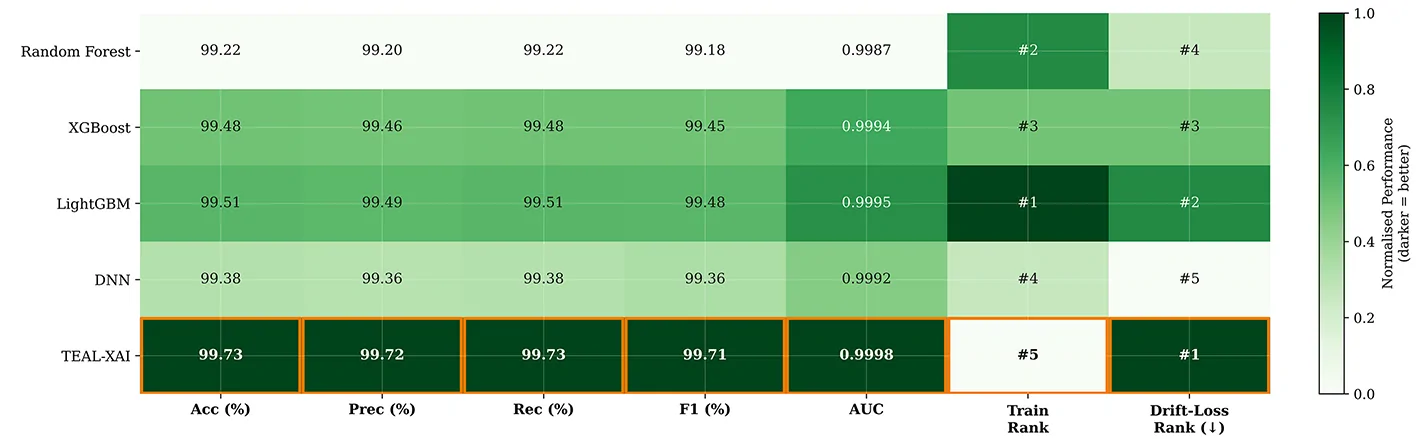
Complete performance summary heatmap—all models × all metrics. Darker green = better performance, rank columns; lower rank # = better efficiency/resilience. TEAL-XAI (highlighted) achieves best classification metrics and highest drift resilience.

## 12 Future study

The future investigations will focus on: (i) online self-adaptive λ estimation via drift severity detection using ADWIN-guided mechanisms; (ii) recurrent and attention-based base learner variants—LSTM, GRU, and Transformer architectures—within the TEAL-XAI adaptive pipeline; (iii) real-time stream evaluation on CICIDS-2017 and CSE-CIC-IDS2018 benchmark streams with documented real-world drift events; (iv) federated learning integration for privacy-preserving distributed IDS deployment; and (v) SHAP importance drift velocity as a proactive, gradient-based drift detection signal replacing explicit statistical change-point detectors.

## Data Availability

Publicly available datasets were analyzed in this study. The KDD Cup 1999 dataset is available at https://kdd.ics.uci.edu/databases/kddcup99/kddcup99.html, the NSL-KDD dataset at https://www.unb.ca/cic/datasets/nsl.html, and the UNSW-NB15 dataset at https://research.unsw.edu.au/projects/unsw-nb15-dataset. Further inquiries can be directed to the corresponding author.
